# LINC00649 underexpression is an adverse prognostic marker in acute myeloid leukemia

**DOI:** 10.1186/s12885-020-07331-0

**Published:** 2020-09-03

**Authors:** Chao Guo, Ya-yue Gao, Qian-qian Ju, Chun-xia Zhang, Ming Gong, Zhen-ling Li

**Affiliations:** Department of Hematology, China-Japan Friendship Hospital, Yinghua East Street, Beijing, China

**Keywords:** Acute myeloid leukemia, Long non-coding RNA, Survival analysis, Gene expression profile

## Abstract

**Background:**

Long noncoding RNAs (lncRNA) play a role in leukemogenesis, maintenance, development, and therapeutic resistance of AML. While few studies have focused on the prognostic significance of LINC00649 in AML, which we aim to investigate in this present study.

**Methods:**

We compared the expression level of LINC00649 between AML patients and healthy controls. The Kaplan-Meier curves of AML patients expressing high versus low level of LINC00649 was performed. The LINC00649 correlated genes/miRNAs/lncRNAs and methylation CpG sites were screened by Pearson correlation analysis with R (version 3.6.0), using TCGA-LAML database. The LINC00649 associated ceRNA network was established using lncBase 2.0 and miRWalk 2.0 online tools, combining results from correlation analysis. Finally, a prediction model was constructed using LASSO-Cox regression.

**Results:**

LINC00649 was underexpressed in bone marrow of AML group than that in healthy control group. The patients of LINC00649-low group have significantly inferior PFS and OS. A total of 154 mRNAs, 31 miRNAs, 28 lncRNAs and 1590 methylated CpG sites were identified to be significantly correlated with LINC00649. Furthermore, the network of ceRNA was established with 6 miRNAs and 122 mRNAs. The Lasso-Cox model fitted OS/PFS to novel prediction models, which integrated clinical factors, ELN risk stratification, mRNA/miRNA expression and methylation profiles. The analysis of time-dependent ROC for our model showed a superior AUC (AUC = 0.916 at 1 year, AUC = 0.916 at 3 years, and AUC = 0.891 at 5 years).

**Conclusions:**

Low expression of LINC00649 is a potential unfavorable prognostic marker for AML patients, which requires the further validation. The analysis by LASSO-COX regression identified a novel comprehensive model with a superior diagnostic utility, which integrated clinical and genetic variables.

## Background

AML is one of most common hematological malignancies, which is characterized by unlimited proliferation of clonal myeloid progenitors and impaired production of normal hematopoietic cells [1]. The prognosis of AML is still heterogenous and unsatisfying: the rate of 5-year survival for AML patients is less than 50% [2], 2-year survival rate of elderly patients is only 20% [3]. Several prediction models have been constructed, among which the updated ELN risk stratification is the most widely used in clinical practice [4], recognizing 3 subgroups of patients according to pretreatment molecular mutations and cytogenetics. Whereas the advances in high-throughput methodology produced multidimensional information on genomes, such as noncoding RNA expression, methylation profile etc.

The size of long noncoding RNAs are generally longer than two hundred nucleotides, and do not have the potential of protein-coding. The interactions of lncRNAs and protein-coding genes are diverse and complex, among which the regulatory mechanism of HOTAIR was well studied in AML. HOTAIR exerts a pro-oncogenic effect in AML, which suppresses p15 expression by methylation of p15 promoter mediated by PRC2, and increases of HOXA5 methylation by directly recruiting DNMT3B [5, 6]. The HOTAIRM1 is another well-studied lncRNA in AML, which played a potential oncogenic role by enhancing expression of HOXA1–4 genes [7]. These results suggest HOXA family genes are important targets of AML-related lncRNAs. HOXA family included 11 genes (HOXA1–7, HOXA9–11 and HOXA13), encoding conserve transcription factors in relation with normal hematopoiesis [8–10]. While dysregulated expression of HOXA family genes is associated with oncogenesis [11].

LINC00649, which we focused in the research, was identified as a prognostic marker in prostate and colorectal cancers by previous bioinformatic analysis [12, 13]. Few studies have investigated the prognostic value of LINC00649 in AML [14]. Notably, the expression of LINC00649 was significantly correlated with HOXA family genes, indicated by the results derived from GEPIA [15] 2.0 online tools (http://gepia2.cancer-pku.cn/) and our own analysis. These results suggest that LINC00649 may be associated with AML survival through regulating HOXA genes.

**Fig. 1:**
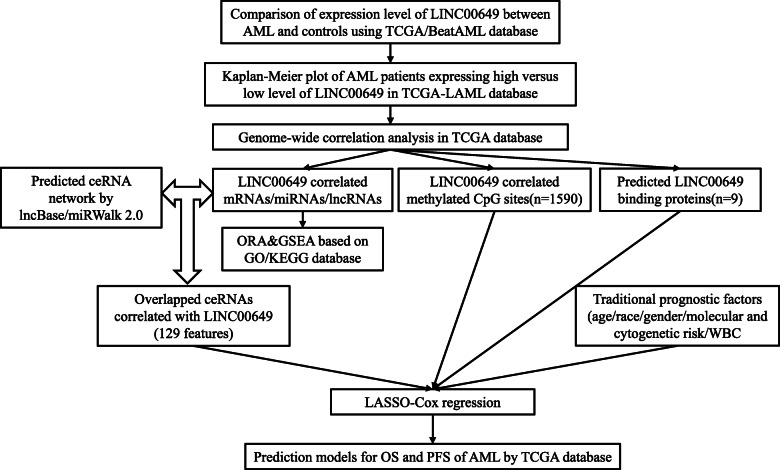
The flow chart of the overall study design. AML, acute myeloid leukemia; TCGA, the Cancer Genome Atlas; OS, overall survival; PFS, progression free survival; LASSO, least absolute shrinkage and selection operator

LncRNA regulated the expression of target genes mainly by the following mechanisms: epigenetic regulation, directly transcription regulation by lncRNA binding proteins, splicing regulation, sponging target miRNAs to form competing endogenous RNA, post-translation regulation [16]. Differentially methylated positions (DMPs) and differentially methylated regions (DMRs) were identified between LINC00649-high and -low expression groups, to reveal the epigenetic changes related to HOXA gene family. Then the possibly binding protein of LINC00649 was uncovered, and its relationship with HOXA genes were conducted. Moreover, we established ceRNA network using overlapped results of prediction by online databases and correlation analysis regarding LINC00649. We did not only aim to reveal the regulating effect of LINC00649 on HOXA genes, but to uncover the association of AML survival with LINC00649 related epigenetic and genetic changes. Therefore, the LASSO regression analysis was employed to fit the survival data of AML patients into the prediction model. An overall flowchart was shown in Fig. 1. Our study presented the rationality to use the expression level of LINC00649 as a prognostic biomarker and established the novel risk model to predict survival of AML cases.

## Methods

### Data source

The RNAseq data was downloaded from BeatAML [17] (http://www.vizome.org/aml) and TCGA database (https://portal.gdc.cancer.gov/), as well as the corresponding clinical and genetic information. The transcriptome dataset in the format of RPKM was obtained from BeatAML database. While the raw count dataset of mRNA and lncRNA was originally obtained from TCGA database, and then transformed to TPM format. The last access to the two database is on 2019.12.25. The GEPIA 2.0 online tool [15] (http://gepia2.cancer-pku.cn) was used to compare expression level of LINC00649 between diverse cancers and corresponding normal tissues to explore the expression signature in AML. The cohorts from BeatAML database were employed to reveal the different expression level of LINC00649 between AML (*n* = 474) and healthy controls (*n* = 33).

### Kaplan-Meier analysis of LINC00649 on AML survival

The AML cohorts from TCGA/BeatAML database were classified as LINC00649-high and LINC00649-low groups respectively, using mean TPM/RPKM of LINC00649. The survival data of AML patients, including OS and PFS, was obtain for TCGA and BeatAML database. Kaplan-Meier analysis were conducted using survival data and log rank test was performed, using *p* value < 0.05 as the cutoff value.

### Identification of LINC00649 binding proteins

The catRAPID is an algorithm predicting RNA-protein paring, by combining hydrogen bond, secondary structure and other inter-molecular factors [18, 19]. The sequence of LINC00649 was downloaded from nucleotide database of PubMed, which was then input into catRAPID omics tools (http://service.tartaglialab.com/). The results were downloaded with predicted protein name and binding sites of protein/RNA.

### Identification of LINC00649 associated protein coding genes/miRNA and lncRNA

The miRNA expression dataset was downloaded from TCGA database, in the format of RPM value. We conducted a correlation analysis between LINC00649 and other protein-coding genes/miRNA/lncRNA by Pearson’s method, using R (version 3.6.0) and cor.test function in stats package. The variables with |Pearson’s coefficient| > 0.4 and *p* value < 0.05 are defined as LINC00649 associated genes. Then to access the enrichment on signaling pathways, we conducted the GSEA based on MSigDB database (http://software.broadinstitute.org/gsea/msigdb) [20–22] using LINC00649 associated gene set and corresponding Pearson’s coefficients. Meanwhile, the ORA was performed based on GO database and KEGG database. The KEGG analysis was conducted by the ClueGO plugin of Cytoscape software (version 3.7.2) and GO analysis by the “topGO” and “REVIGO” package and R (version 3.6.0).

### Identification of LINC00649 associated methylation prolife

To investigate the methylation signature in relation to LINC00649 expression, we obtained normalized beta value matrix for AML cohort from TCGA database (146 AML patients, Illumina Infinium HumanMethylation450 platform). The correlation analysis was performed between LINC00649 expression and methylation status (beta value) of individual methylation position and methylation regions.

### Establishment of LINC00649-centric competitive endogenous RNA (ceRNA) network

The predicted target miRNA set of LINC00649 was obtained by prediction module of lncBase v2 database, which is based on the microRNA/lncRNA target predicting algorithm [23] (http://carolina.imis.athena-innovation.gr/diana_tools/web). The target mRNAs, the 3′ UTR of which were predicted to bind the identified miRNAs, were screened by miRWalk 2.0 online tools [22, 24] (http://zmf.umm.uni-heidelberg.de/). We defined the LINC00649-centric ceRNAs as intersection between predicted target miRNA set and LINC00649-associated miRNA set generated from previous correlation analysis.

### Establishment of prediction model for AML survival

In above analysis, we identified the LINC00649-centric ceRNA network and methylation changes in relevance of LINC00649, which were supposed to be key elements linking to prognosis of AML. Moreover, to improve the prognostic model, we constructed a multidimensional survival analysis, integrating clinical features, expression level of LINC00649 and related mRNAs/miRNAs in ceRNA network, and methylation status of correlated CpG sites. We downloaded expression dataset (RNA-seq data and miRNAseq data), methylation dataset (beta value), from TCGA database (https://portal.gdc.cancer.gov/). Because the APL patients, also known as M3 type in FAB classification, received the quite different treatment and have more superior survival than other types of AML, we excluded such patients from AML cohort. The traditional clinical variables were taken into analysis, including age, race, gender, risk stratification based on molecular/cytogenetic signature, and counts of white blood cells. According to ELN2017 recommendations [4], AML patients were classified as ‘good’, ‘intermediate’ and ‘poor’ groups, based on the karyotype and gene variation. Finally, 124 AML patients with the intact data were included in our survival analysis. The PFS and OS were fitted to LASSO-Cox model, establishing a completely novel prognostic model for AML patients. The glmnet package were implemented for LASSO regression analysis, which penalized the variables to eliminate less informative predictors, resulting in more interpretable and simpler models. The final coefficient for each variable included in the model, was the average value of the coefficient estimates obtained for the set of cross-validation evaluations. To compare continuous variables between groups, we conducted the Wilcoxon rank-sum test. The Fisher exact test was employed for testing the correlation between categorical variables between groups.

## Results

### LINC00649 is under-expressed in bone marrow of AML

**Fig. 2:**
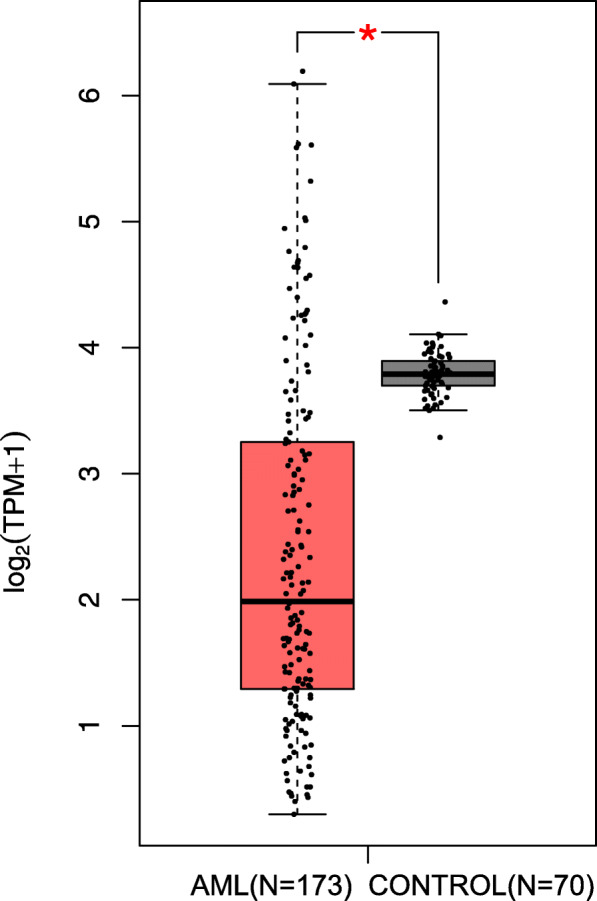
The comparison of expression level for LINC00649 between TCGA-AML cohort (*N* = 173) and GTEx normal bone marrow samples (*n* = 70) using GraphPad Prism (version 7.0)

**Fig. 3:**
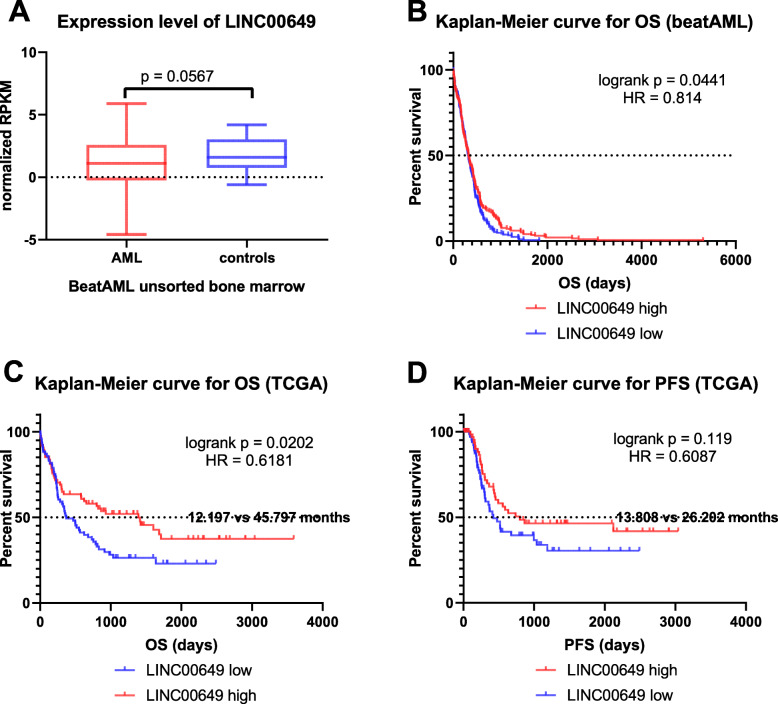
relevance of LINC00649 to AML, expression data of LINC00649 in bone marrow of AML patients versus healthy controls in BeatAML database (**a**). From TCGA and BeatAML database, the Kaplan-Meier curves of AML patients whose bone marrow cells express high versus low level of LINC00649, for OS (**b** & **c**) and PFS (**d**). The prognosis of LINC00649-low group is more unfavorable than that of LINC00649-high group. These results were generated using R software (version 3.6.0)

Using GEPIA 2.0 database [15], the comparison of LINC00649 expression levels (TPM) between the tumors and normal tissues across multi-cancer types, derived from TCGA and GTEx database, was shown in Fig. 2. The expression of LINC00649 in normal hematopoietic cells is the highest among all included cancers and tissues (TPM value = 12.83). Moreover, the expression of LINC00649 is much lower in AML cells in comparison with normal hematopoietic cells (TPM value 2.96 vs 12.83), which is converse in most of other cancer types. While in BeatAML database, AML patients also have a trend of lower expression of LINC00649 than that of healthy controls (*p* = 0.0567, Fig. 3a).

### Expression of LINC00649 is correlated with survival of AML patients

The clinical/genetic features of LINC00649-low and LINC00649-high group were described in Table 1. LINC00649-low patients were associated more unfavorable cytogenetic and molecular risk profiles (*p* = 0.001) and higher WBC counts (*p* = 0.001).

**Table 1:** The comparison of clinical and genetic features between LINC00649-high and LINC00649-low groups. A total of 145 patients from TCGA and 451 patients form BeatAML database were included. The molecular and cytogenetic risk of LINC00649-low group is more adverse than that of LINC00649-high group (*p* < 0.001)

	TCGA			beatAML		
	LINC00649-low group (*n* = 72)	LINC00649-high group (*n* = 73)	*p* value	LINC00649-low group (*n* = 225)	LINC00649-high group (*n* = 226)	*p* value
**Age (year)**	55.21 ± 2.011	54.47 ± 2.137	0.81	59.81 ± 1.055	53.62 ± 1.356	0.049
**Gender**			0.589			0.775
Female	27	31		95	98	
Male	35	31		130	127	
**Race**			0.838			0.801
White	47	45		39	37	
Other races	15	17		186	188	
**Mutation count**	9.82 ± 0.624	9.77 ± 0.803	0.962	NA	NA	
**Risk stratification of cytogenetics**			0.001			NA
Good	0	15		NA	NA	
Intermediate	48	28		NA	NA	
Poor	14	19		NA	NA	
**Risk stratification of molecular mutation**			0.001			0.002
Good	0	15		99	70	
Intermediate	45	28		44	73	
Poor	17	19		82	82	
**WBC**	47.498 ± 5.091	22.705 ± 5.040	0.001	34.807 ± 2.979	27.615 ± 3.465	0.112

The PFS and OS were significantly inferior for LINC00649-low group in comparison with LINC00649-high group (Fig. 3c & d). The median OS of LINC00649-high and LINC00649-low groups are 45.797 versus 12.197 months, and *p* value of log rank test is 0.0202 (Fig. 3c). The median PFS of LINC00649- low and LINC00649- high groups are 26.202 versus 13.808 months respectively, and *p* value of log rank test is 0.119 (Fig. 3d). These results are consistent with OS analysis using BeatAML database (Fig. 3b).

### Prediction of LINC00649 binding proteins

The predicted proteins were listed in Supplementary Table 1. A total of 120 binding sites involving 9 proteins were identified (ELAVL2/TIAL1/PTBP1/CELF1/SRSF9/SRSF2/SRSF3/ESRP2/RBFOX2). Then correlation analysis of predicted proteins and HOXA genes was conducted using RNA-seq data of TCGA database (Table 2), where the significantly correlated gene pairs were colored in red. The TIAL1, SRSF9, SRSF2, SRSF3 and RBFOX2 were significantly correlated with the expression of HOXA genes (*p* < 0.05)

**Table 2:**
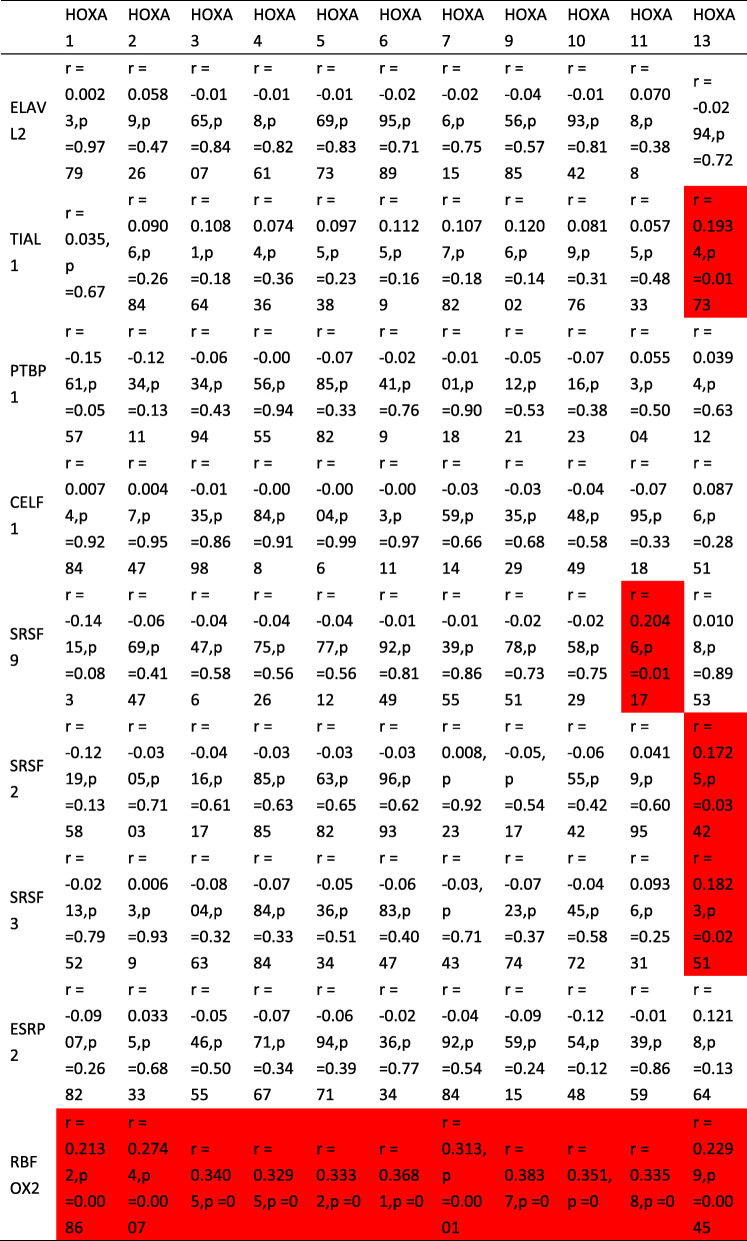
The results of Pearson’s correlation analysis between predicted LINC00649 binding proteins and HOXA genes. The significant correlated pairs were colored in red

### Identification of LINC00649 associated protein coding genes/miRNAs and lncRNAs

**Fig. 4:**
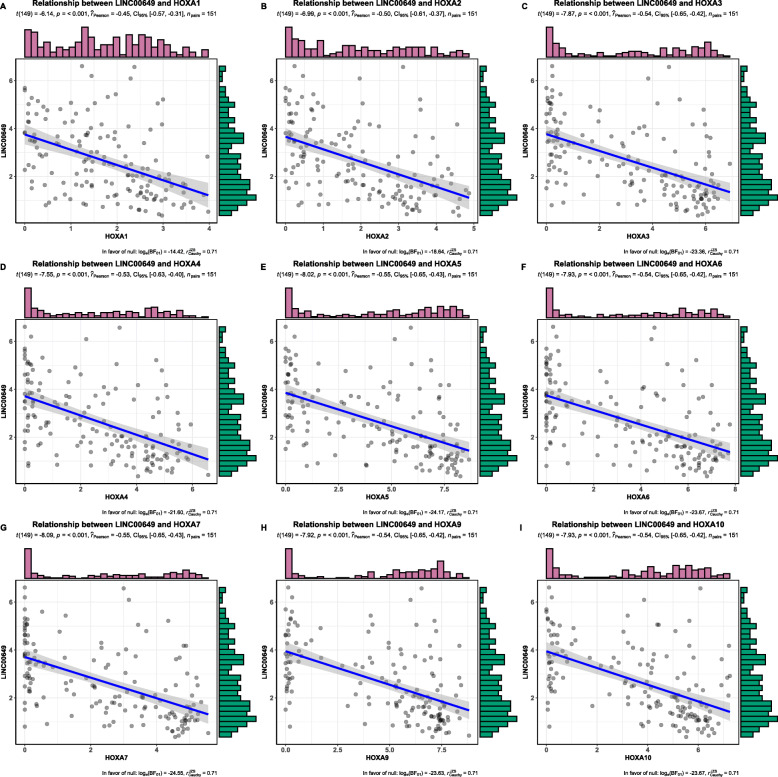
The Pearson correlation and linear regression for LINC00649 and HOXA family genes by R software (version 3.6.0)

In total, 154 protein-coding genes, 28 lncRNAs and 31 miRNAs were identified to be significantly correlated with LINC00649 expression level (Supplementary Table 2). The expression of 9 HOXA family members (HOXA1/2/3/4/5/6/7/9/10) was negatively correlated with LINC00649 significantly (Fig. 4), indicating that LINC00649 involves in downregulation of HOXA genes.

**Fig. 5:**
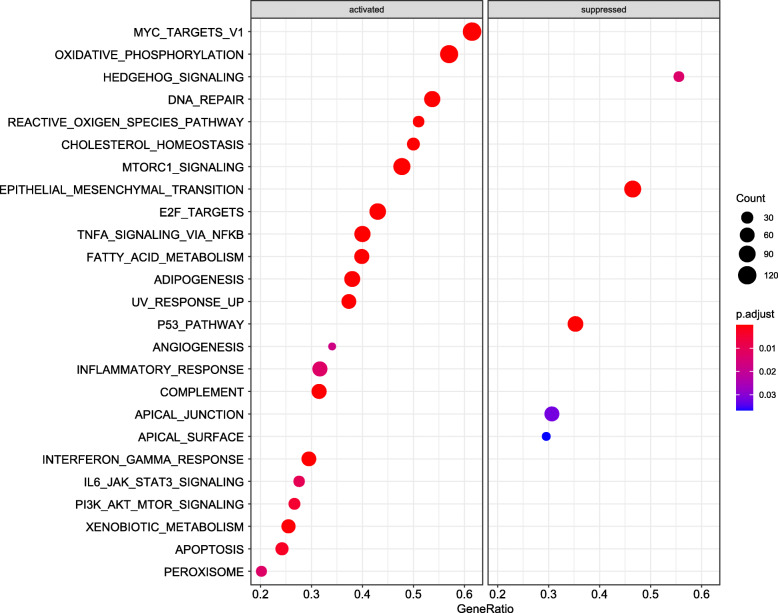
The dotplot of GESA results for LINC00649 correlated genes by R software (version 3.6.0). The size of dots stands for gene counts in the specific pathway, and the color represents correlated with adjusted *p* value. Nineteen activated and 6 suppressed pathways were identified

**Fig. 6:**
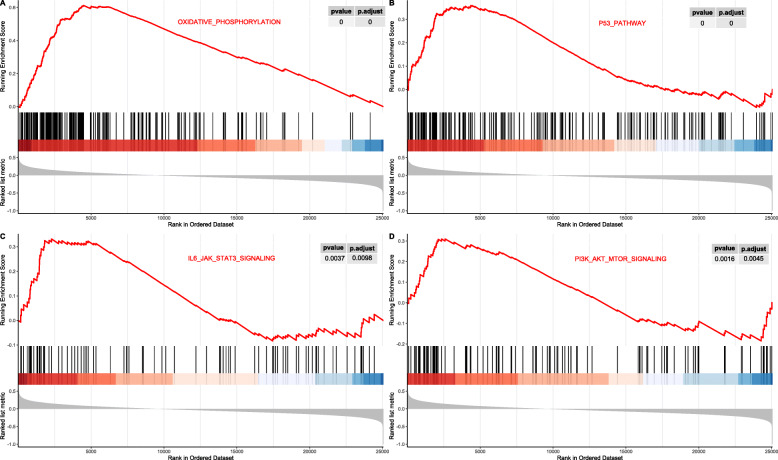
The running enrichment score curve for oxidative phosphorylation, p53 signaling, IL-6/JAK/STAT3, PI3K/Akt/mTOR generated by R software (version 3.6.0)

The results of GESA indicated that under-expression of LINC00649 was associated with activation of 19 pathways and suppression of 6 pathways (Fig. 5). The activated pathways included oxidative phosphorylation, IL6-JAK-STAT3 signaling, PI3K-Akt-mTOR signaling (Fig. 6), angiogenesis, etc. while the suppressed pathways included P53 pathway, Hedgehog signaling, epithelial mesenchymal transition, etc. (Fig. 6).

**Fig. 7:**
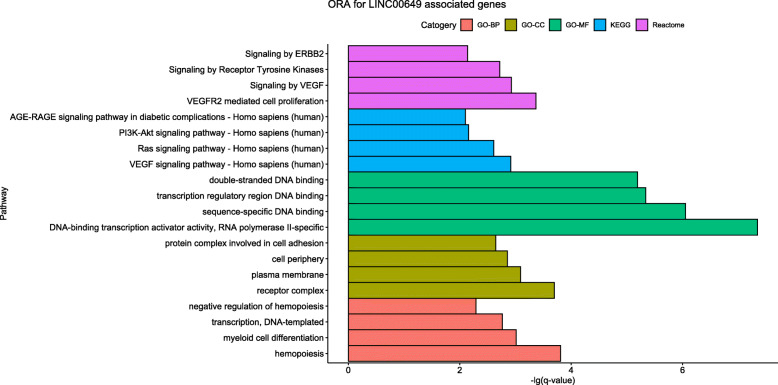
The dotplot of ORA (GO/KEGG/Reactome analysis) results for LINC00649 correlated genes by R software (version 3.6.0). The size of dots stands for gene counts in the specific pathway, and the color represents correlated with adjusted *p* value

For the results of ORA (Fig. 7), the following biological processes were identified by GO enrichment analysis: negative regulation of hematopoiesis, DNA-templated transcription, myeloid cell differentiation, etc. The cell components of LINC00649 associated genes were enriched in protein complex involved in cell adhesion, cell periphery, plasma membrane, etc. The molecular function enriched by LINC00649-associated genes were double-stranded DNA binding, transcription regulatory region DNA binding, sequence-specific DNA binding, etc. Based on the KEGG database, LINC00649 associated genes were enriched in PI3K-Akt signaling pathway, Ras signaling pathway, etc. The analysis based on Reactome database indicated that these genes were enriched in Signaling by ERBB2, Signaling by Receptor Tyrosine Kinases, Signaling by VEGF, etc.

### Identification of LINC00649 associated methylation prolife

One thousand five hundred ninety methylated CpG sites were identified to be significantly correlated with LINC00649 expression (*p* < 0.05, |r| > 0.4), listed in Supplementary Table 3), among which 7 methylation positions involving with HOXA6/HOXA9/HOXA10 (cg21172377, cg07483304, cg05490659, cg02000808, cg19816811, cg16880946, cg18931036) have prognostic significance for OS, based on TCGA database using MethSurv online tools [25] (https://biit.cs.ut.ee/methsurv/). Therefore, we inferred that the similar methylation modulation of HOTAIR on HOXA5, may be related to regulation of HOXA genes by LINC00649 [6]. In the following construction of prediction model, the methylation level of 1590 positions were included in initial LASSO analysis.

### The competitive endogenous RNA network of LINC00649

**Fig. 8:**
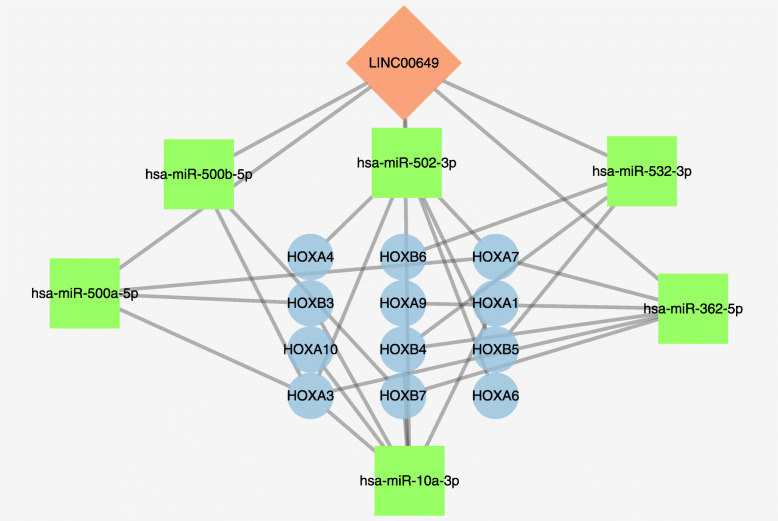
The ceRNA network of LINC00649 related with HOX family genes by Cytoscape (version 3.7.2)

Six miRNAs and 122 mRNAs were included in LINC00649 centric ceRNA network (Supplementary Table 4). The miR-10a-3p, miR-500a-5p, miR-500b-5p, miR-532-3p, miR-502-3p and miR-362-5p were both predicted as sponging miRNAs and statistically significantly correlated with LINC00649 expression (Fig. 8). Notably, these miRNAs are predicted to negatively inhibit the expression of HOX family genes, suggesting LINC00649 may exert biological effect through sponging miRNAs.

### Establishment of the prediction model

A LASSO-COX regression analysis was conducted to identify the prediction models fitting AML OS/PFS, which initially included age, ELN2017 risk stratification, expression level of elements in the LINC00649-centric ceRNA network, and methylation status of LINC00649-associated CpG sites. After 1000 times of iteration, the developing process of model screened the optimal risk scores using summation of selected variables weighted by coefficients (Table 3). The ‘cutoffROC’ package was employed to determine the cut-off value for OS and PFS model respectively, based on the time-dependent ROC curves. Then AML cohort from TCGA database, was divided into high-risk group and low risk group, by the cutoff value.

**Table 3:** The variables and coefficients of prediction models for OS and PFS. The risk score for individual patients was calculated as summation of each variable and corresponding coefficient

OS		DFS	
Variables	Coefficients	Variables	Coefficients
Age	0.01517872	HSDL1	−0.069116277
Risk_moleuclar	0.08487131	KIF26A	−0.105321193
KIF26A	− 0.06888634	ZNF124	− 0.027336956
SERINC5	−0.34892134	LPAR5	0.183893293
EVPL	0.01905074	PATE2	−0.056221659
SMAGP	0.04229574	hsa-miR-502-3p	0.141651616
CD320	0.07386408	cg07613391	−0.05203838
hsa-miR-502-3p	0.04133291	cg23495279	0.174167913
cg27456487	0.10692372	cg06637001	−0.143845051
cg15440158	−0.20311719	cg02942845	−0.181993998
cg21760402	−0.32770473	cg00081084	5.458822697
cg00081084	1.0182775	cg10520887	−0.129876909
cg14533068	0.13680058	cg13331200	0.568422555
cg18597188	0.1459364	cg00599124	0.082618413
cg22291265	−0.17893264	cg21347874	−0.358914855
cg13475665	−0.05682765	cg14459021	0.079482707
cg06812991	−0.29442296	cg20386404	−0.013977599
cg02057391	−0.38221762	cg27100436	0.563627584
cg14459021	0.35068618	cg21844856	−1.256126321
cg20386404	−0.09889255		
cg05140293	0.03990318		
cg10152449	−0.73415156		
cg16280141	1.19906562		
cg15275758	−0.65571266		

**Fig. 9:**
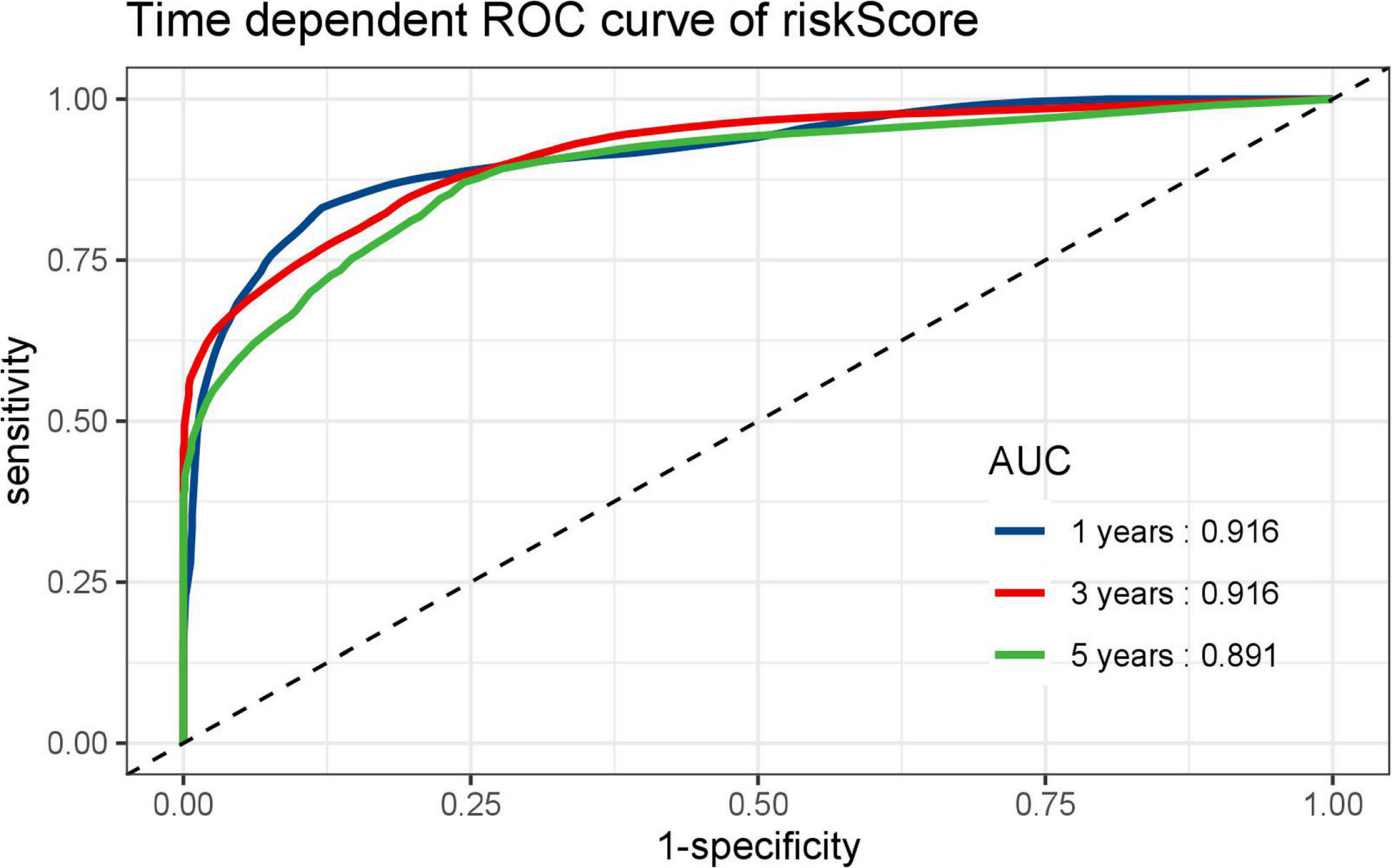
The time-dependent ROC curves of risk score by our prediction model for OS by R software (version 3.6.0)

**Fig. 10:**
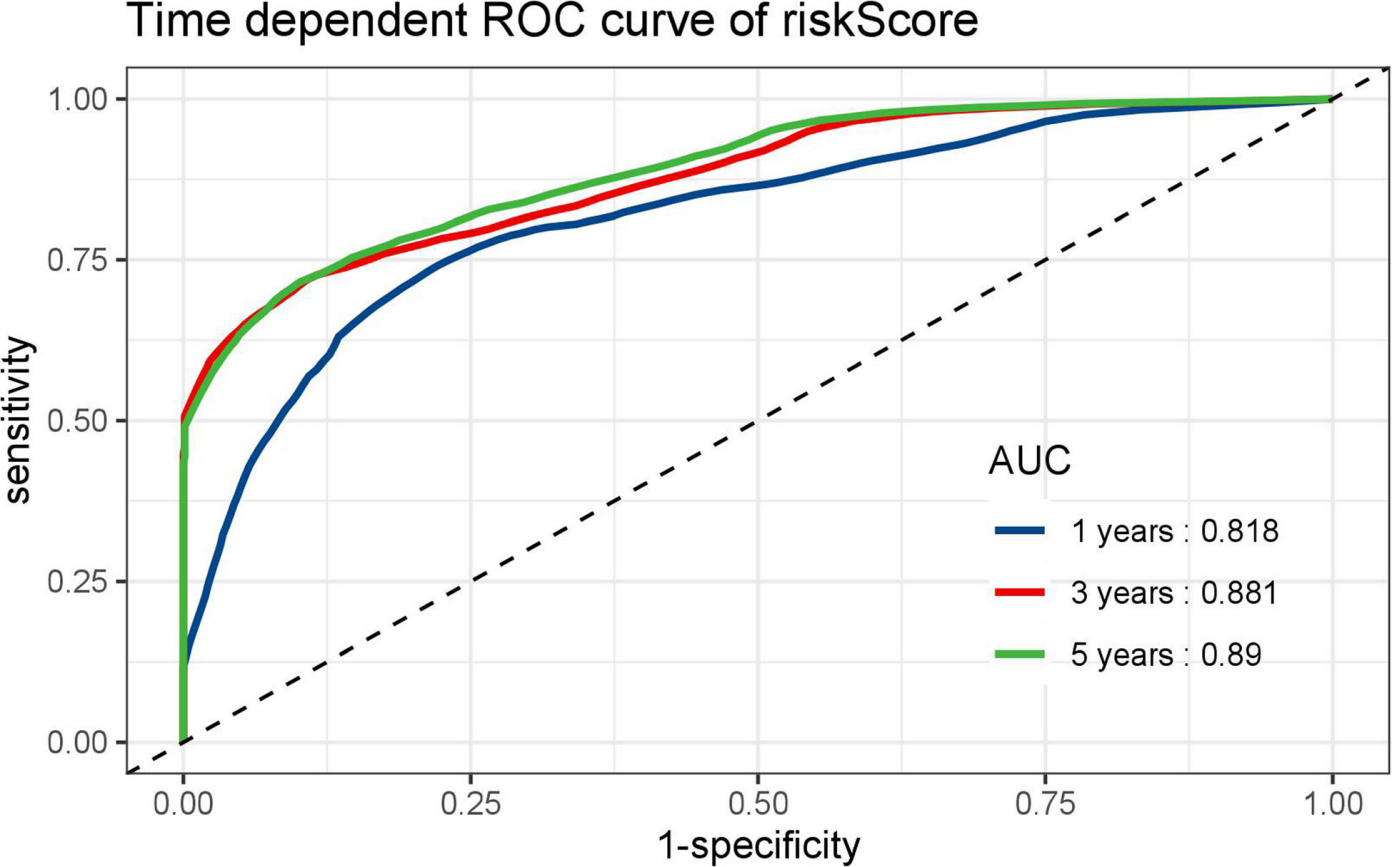
The time-dependent ROC curves of risk score by our prediction model for PFS by R software (version 3.6.0)

**Fig. 11:**
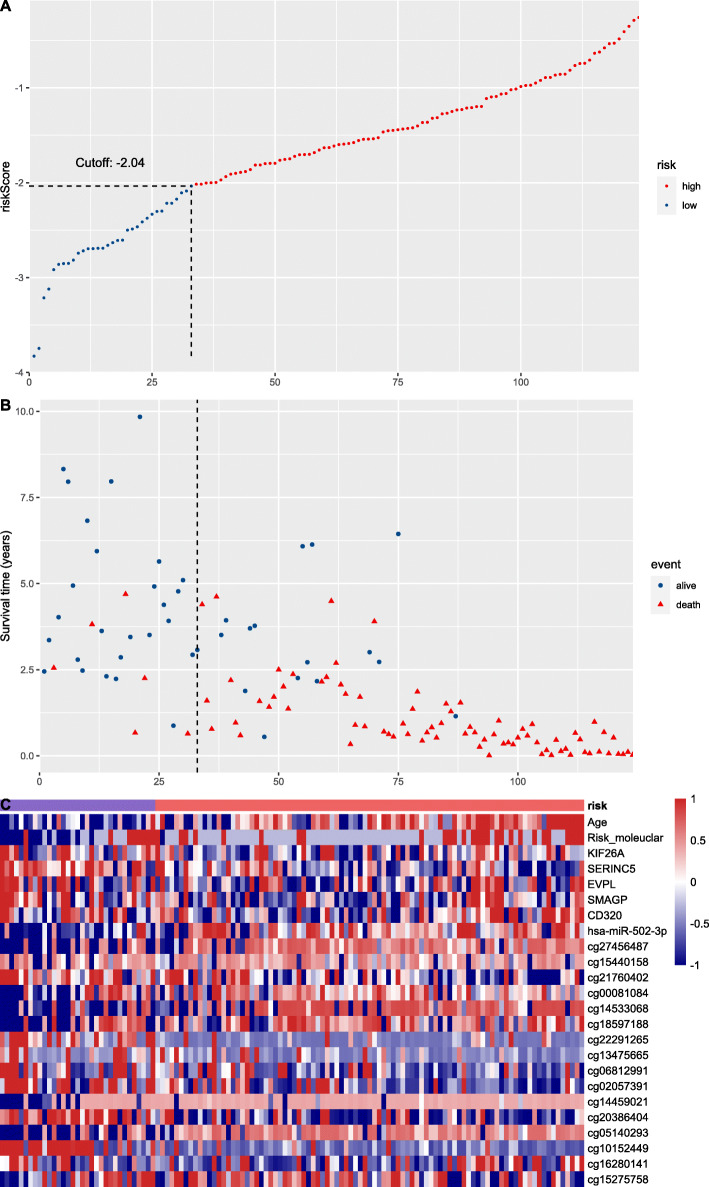
The distribution of risk score (**a**), patients’ survival events (**b**), and risk to variable heatmap (**c**) for OS predicting model by R software (version 3.6.0), the X-axis stands for individual AML patients ordered by ascending risk score

**Fig. 12:**
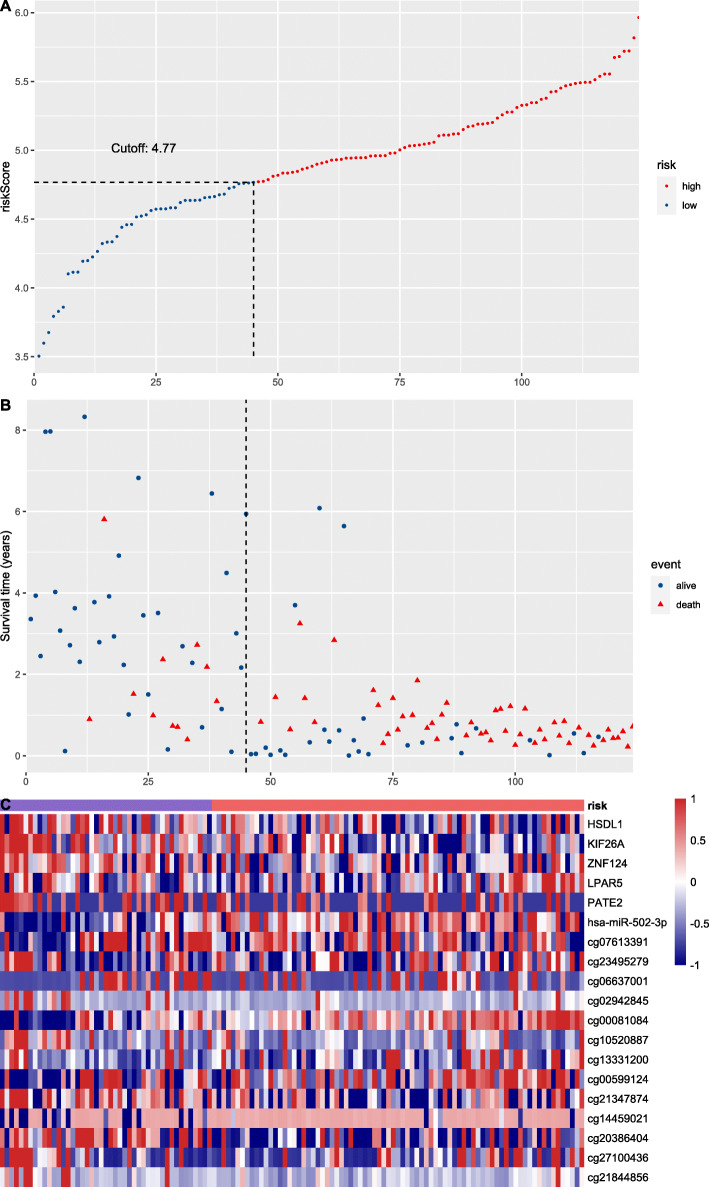
The distribution of risk score (**a**), patients’ survival events (**b**), and risk to variable heatmap (**c**) for PFS predicting model by R software (version 3.6.0), the X-axis stands for individual AML patients ordered by ascending risk score

**Fig. 13:**
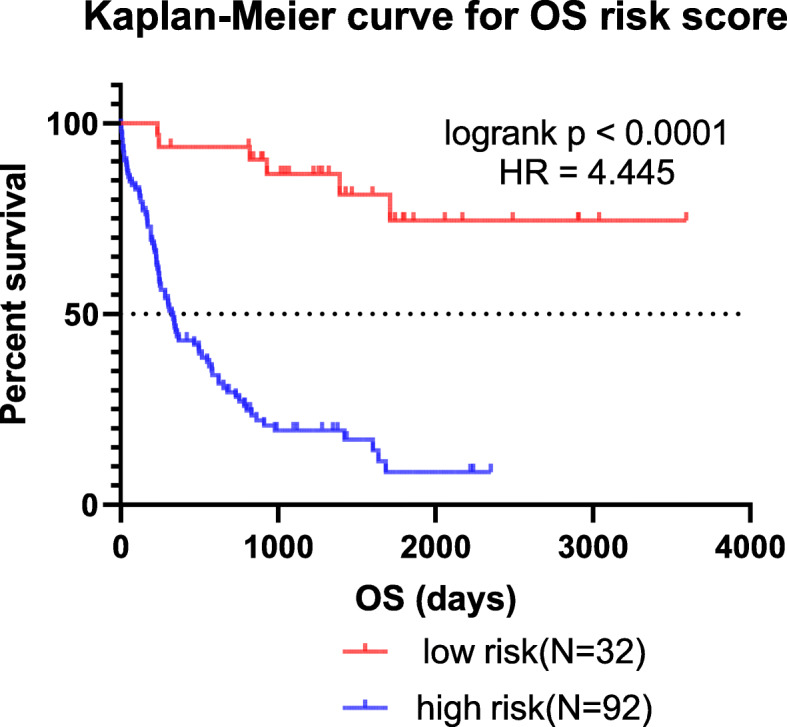
The Kaplan-Meier OS curves of AML patients for low and high-risk group dichotomized by risk score of our prediction model, which is generated by GraphPad Prism (version 7.0)

**Fig. 14:**
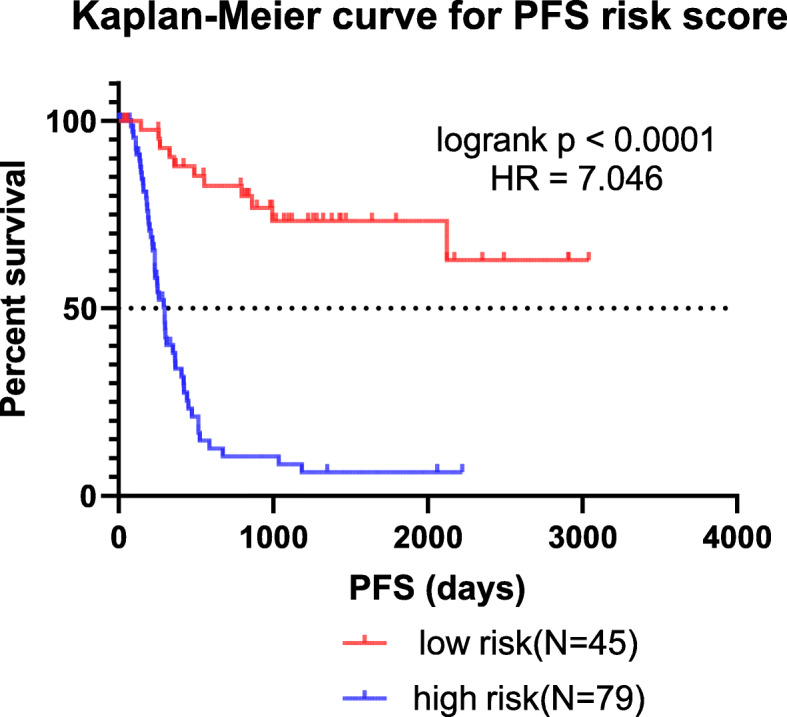
Kaplan-Meier PFS curves of AML patients for low and high-risk group dichotomized by risk score of our prediction model, which is generated by GraphPad Prism (version 7.0)

The AUC at 1/3/5-year AUC of the prediction model are 0.916/0.916/0.891 respectively for OS (Fig. 9), and 0.818/0.881/0.89 for PFS (Fig. 10). The distribution of risk scores, survival-events plots and the heatmap of variables for individual patients were shown in Figs. 11 & 12 for OS and PFS, respectively. Then Kaplan-Meier plot was employed to elucidate the difference of survival between high risk and low risk group (Figs. 13 & 14). The median OS and PFS of low-risk group were not reached, which were much better than the high-risk group. The results of Kaplan-Meier plots implicated that the novel prediction model was efficient for selecting AML patients with superior prognosis. The performance of our model is encouraging, while further prospective research is needed to evaluate the diagnostic value of this model more precisely.

## Discussion

The long noncoding RNAs have been uncovered to exert an pivotal influence on cell proliferation and apoptosis of AML, the mechanisms of which include altering methylation status of gene promoters [5, 6], recruiting epigenetic complex on gene promoters [26], reshaping chromatin [27, 28], sponging miRNAs to regulate gene expression [29–32], etc. HOTAIR is one of the most studied lncRNAs in AML, which is upregulated in de novo AML patients and predicts an adverse prognosis [33]. HOTAIR locates in HOXC gene cluster on chromosome 12 and exerts biological effect through modulating HOXA family genes. Intriguingly, through analysis of TCGA expression data, we found LINC00649 was also correlated with most of HOXA family genes, which encoded crucial transcription factors in normal hematopoiesis, pathogenesis of AML and resistance to chemotherapy [34–36]. In comparison with healthy controls, AML patients have aberrantly lower LINC00649 expression in our results. Meanwhile, for most of cancers other than AML, expression level of LINC00649 in cancer cells is higher than that of corresponding normal tissues. Furthermore, the survival (OS and PFS), of LINC00649-low group, was significantly worse than that of LINC00649-high group. The unusual expression signature and prognostic value of LINC00649 drove us to explore the possible molecular mechanisms and uncover its biological function.

According to catRAPID algorithm, 9 proteins containing 120 sites were identified to be potentially binding to LINC00649. TIAL1, SRSF9, SRSF2, SRSF3 and RBFOX2 were identified to be associated with HOXA gene expression. TIAL1 is the RNA binding protein, which binds to target sites and splice the pre-mRNA alternatively [37, 38]. SRSF9 involves in constitutive mRNA splicing and can modulate the target of alternative splicing [39]. SRSF9 was reported to be involved in the cell proliferation and apoptosis in bladder and cervical cancer [40, 41], and related to prognostic alternative splicing events of renal clear cell carcinoma [42]. SRSF2 and SRSF3 are also splicing factors, which belong to serine/arginine-rich protein family. Functional mutations of SRSF2 drive the cancer genesis of hematopoietic cells [43]. SRSF3 is a multiple cancer related splicing factor, namely glioblastoma [44], colon cancer [45], oral squamous carcinoma [46], etc. Moreover, the expression of SRSF2/3 is significantly decreased in de novo AML patients in comparison with that of healthy controls. RBFOX2 can bind to 5′- UGCAUGU-3′ element of target RNA, exerting alternative splicing. RBFOX2 can modulate erythropoiesis, by promoting alternative selection of exon 16 in protein 4.1R, the product of which is essential for erythrocyte membrane stability [47, 48]. Notably, the expression of RBFOX2 is significantly correlated with all members of HOXA family genes (Supplementary Figure 1), suggesting potential interaction between them. Furthermore, the pancancer-TCGA expression data was download from UCSC database (https://xenabrowser.net/hub/), the correlation of RBFOX2 and HOXA genes was analyzed by Pearson’s method (Supplementary Figure 2-1/2/3). Notably, the significant association of RBFOX2 and HOXA is a common feature among cancers generated from different tissues. The expression dataset of normal tissue was downloaded from GTEx database (https://www.gtexportal.org/home/), similar analysis showed that the correlation is insignificant in normal bone marrow (Supplementary Figure 3-1/2/3), which indicated the relationship was a disease-specific feature for AML instead of normal hematopoiesis. All 5 splicing factors and LINC00649 are potential co-regulators for HOXA genes in AML, which has not been explored before.

Based on the results of GESA, the upregulation of PI3K-Akt-mTOR signaling, IL6-JAK-STAT3 signaling, oxidative phosphorylation was identified in LINC00649-low group. The activation of PI3K-Akt-mTOR signaling were found in 50% of AML patients [49, 50]. The PI3K-Akt signaling controls leukemic blast cells proliferation and clonogenicity [51, 52]. Aberrantly functional receptor tyrosine kinases drive the activation of PI3K-Akt-mTOR pathway, including IGF1/IGF1R [53, 54], activated FLT3 [55] and DEK-NUP14 fusion protein [56]. The inhibitors of PI3K-Akt-mTOR axis have shown preliminary anti-leukemia effects against AML both in vivo and in vitro [57–63]. The IL6-JAK-STAT3 pathway plays a crucial role in oncogenesis of diverse cancers [64]. Constitutive phosphorylation of STAT3 by autocrine secretion of IL6 is revealed in AML cells [65]. Activation of STAT3 is also uncovered in pediatric AML samples, and the small-molecule inhibitor of STAT3 can induce apoptosis and inhibit formation of blast colonies in vitro [66]. The maintenance of leukemia stem cells depends on BCL2 mediated oxidative respiration, instead of glycolysis as in normal hematopoietic cells [67]. The metformin, targeting oxidative phosphorylation (OXPHOS), induces apoptosis of human leukemia cells in an AMPK-independent way [68]. Cytarabine resistant leukemia cells are characterized by activated OXPHOS, with the high level of reactive oxygen species. Additionally, the resistance can be reversed by agents inducing low OXPHOS status [69]. The p53 signaling and Hedgehog signaling were found to be suppressed in GSEA. Non-mutational p53 dysfunction was common in AML and implicated in diverse inactivating mechanisms [70]. Dysregulation and activation of PI3K-Akt-mTOR signaling pathway can activate MDM2 and interact with NF-kappaB signaling pathway, leading to dysfunction of p53 [71]. The activation of PI3K pathway was revealed in LINC00649-low group, which may cause the suppression of p53 signaling and inferior survival considering the central role of p53 in the complex network of AML-associated signaling pathway.

The KEGG analysis showed that the LINC00649-associated genes were enriched in AGE-RAGE, PI3K-Akt, Ras and VEGFR signaling pathways. The AGE and RAGE signaling has been studied in AML, which indicated AGE activated MAP kinase, PI3K and JAK/STAT pathway, leading to proliferation of primary AML samples and AML cell lines [72]. Activation of Ras signaling can also promote the dysfunction of p53 by similar mechanism of PI3K-Akt signaling [71]. VEGFR is reported to be overexpressed [73] in AML, which is in accordance with our results. The activated VEGFR signaling promoted the proliferation, survival and resistance to chemotherapy of AML blasts [74]. VEGFR targeting therapy has been developing and showing preliminary benefit for AML in vitro [75–77]. While the Reactome analysis demonstrated other enriched pathways, including signaling by ERBB2 and VEGFR2 mediated cell proliferation. Mudritinib, an ERBB2 inhibitor, was reported to eliminate AML cell both in vivo and in vitro [78] VEGFR2 is a ‘hot’ target in AML, and relevant to chemotherapy-sensitivity, pro-survival effect and angiogenesis in bone marrow [79, 80]. VEGFR2-targeting therapy is being developed in preclinical stage [80, 81]. The dysregulation of all above pathways contributed to the difference of survival between LINC00649-high and low groups.

Furthermore, HOXA family genes methylation status was shown to be correlated with LINC00649. The methylation status of seven CpG sites involving with HOXA6/HOXA9/HOXA10 was correlated with expression of LINC00649. Notably, all involved sites were of significance for AML overall survival (Supplementary Figure 4). Considering that lncRNA HOTAIR can modulate the methylation status of HOXA5 by inhibiting DNMT3B [6], our results suggested similar epigenetic mechanism may implicated in the regulation of HOXA genes.

To improve the diagnostic utility, we brought in multi-dimension information to establish a prediction model on AML survival. The traditional prognostic factors (age, gender, ELN2017 risk stratification, etc) and the associated expression data (predicted LINC00649 binding proteins, miRNAs/mRNAs in the ceRNA network), and methylation data (altered methylated CpG sites) were included into the prediction model, by which the OS and PFS data were fitted into using the LASSO regression analysis. A few prediction models, including genetic information of AML patients, have been developed previously, including Clinseq-G [82] (AUC for 3-year OS is 0.730), ELN2017 stratification in the validation cohort [82] (AUC for 3-year OS is 0.65), Li Z et al. [82] (AUC for 3-year OS is 0.70), Huang R et al. [83] (AUC for 1 year OS is 0.666, AUC for 5 year OS is 0.707), Ha M et al. [84] (AUC for 5-year OS is 0.613). The AUC of our prediction models is better than all these models, possibly attributing to the integrated multi-dimension information. On the other hand, the Kaplan-Meier plots supported the risk stratification using our models to divide patients into high-risk and low-risk group, which well recognized the patients with much better prognosis (the median OS of low-risk group has not reached). While due to lack of integrated information in one cohort like TCGA database, which included clinical/RNAseq (protein-coding and noncoding RNA)/miRNAseq/methylation datasets, we can hardly validate this model independently. However, the present work brings clues and insights to further studies, by providing potential biomarker and therapeutic targets.

In our OS-prediction model, novel markers were identified (Table 3). EVPL is a component of the cornified envelope of keratinocytes, the genetic variations of which are associated with several solid cancer types [85–89]. While the association of other protein-coding genes or noncoding RNAswith either hematological or solid malignancy has not been investigated. Among the included methylation positions, individual methylation status of cg27456487 (MPO), cg05140293 (TTLL4), cg10152449 (CHST12), cg22291265 (SHANK1), cg18597188 (XRCC3), cg14533068 (SYNJ2), cg00081084 (TBCB) and cg20386404 (PTPN14) were significantly associated with AML survival, according to MethSurv online tools (https://biit.cs.ut.ee/methsurv/) (Supplementary Figure 5). A low expression ratio of MPO has been reported as a deleterious marker for AML, indicating a lower complete remission rate [90] and shorter PFS [91]. In untreated AML patients, hypermethylation status of MPO is detected and correlates with MPO expression, which can be induced by demethylating agents [92]. The alteration of MPO is demonstrated as an indicator for DNA methylation pattern implicating downregulation of DNMT3B [93], our results supported its significance in pathogenesis of AML. Other included methylated sites have not been reported to implicated in AML. In PFS prediction model (Table 3), no genetic variables were described in relation with AML previously. Notably, KIF26A was included in both OS and PFS model, which belongs to kinesin superfamily and is reported as an oncogenic marker for breast cancer [94] and pancreatic ductal carcinoma [95].

## Conclusion

To the best of our knowledge, this is the first research to demonstrate the under-expression of LINC00649 is a potential unfavorable prognostic indicator for AML. Additionally, the novel multi-dimensional prediction models were established with superior diagnostic utility. Further studies are needed on the precise molecular mechanisms and validation of data analysis.

## Supplementary information

**Additional file 1: Table S1.** The results of predicted binding proteins of LINC00649 by catRAPID algorithm.

**Additional file 2: Table S2.** The results of genome-wide expression correlation analysis of LINC00649, for protein-coding genes/miRNAs/lncRNAs respectively (*p* value < 0.05 and |r| > 0.4).

**Additional file 3: Table S3.** The results of genome-wide methylation correlation analysis of LINC00649, showing the significantly correlated methylated positions.

**Additional file 4: Table S4.** the identified lncRNA, miRNA and mRNA in the ceRNA network.

**Additional file 5: Figure S1.** The results of correlation analysis of RBFOX2 and HOXA genes by R software (version 3.6.0).

**Additional file 6: Figure S2.** Pan-cancer correlation analysis of RBFOX2 and HOXA genes by R software (version 3.6.0). The X axis stands for -log_10_(*p* value), while the Y axis represent Pearson coefficients. The purple dots in the right upper quadrant represent cancer types, in which the correlation is significant and positive. The result in AML was red dots and annotated by text.

**Additional file 7: Figure S3.** pan-tissue correlation analysis of RBFOX2 and HOXA genes by R software (version 3.6.0). The X axis stands for -log_10_(*p* value), while the Y axis represent Pearson coefficients. The purple dots in the right upper quadrant represent normal tissue types, in which the correlation is significant and positive. The result in normal bone marrow was red dots and annotated by text.

**Additional file 8: Figure S4.** The results of Kaplan-Meier analysis obtained by MethSurv online database.

**Additional file 9: Figure S5.** The results of Kaplan-Meier analysis obtained by MethSurv online database.

## Data Availability

The data that support the findings of this study are available from BeatAML database (http://www.vizome.org/aml) and TCGA database (https://portal.gdc.cancer.gov/), which are all publicly available.

## Abbreviations

AML: Acute myeloid leukemia
ELN: European LeukemiaNet
LINC00649: Long Intergenic Non-Protein Coding RNA 649
HOTAIR: HOX Transcription Antisense RNA
HOTAIRM1: HOXA Transcript Antisense RNA, Myeloid-Specific 1
HOXA: Homeobox protein HOX cluster A
GEPIA: Gene Expression Profiling Interactive Analysis
DMP: Differentially methylated positions
DMR: Differentially methylated regions
ceRNA: Competing endogenous RNA
TCGA: The Cancer Genome Atlas
OS: Overall survival
RPKM: Reads Per Kilobase Million
TPM: Per Kilobase Million
PFS: Progression free survival
KEGG: Kyoto Encyclopedia of Genes and Genomes
GO: Gene ontology
LASSO: Least absolute shrinkage and selection operator
GESA: Gene set enrichment analysis
ORA: Over-representation analysis
APL: Acute promyelocytic leukemia
TIAL1: Nucleolysin TIAR
SRSF9: Serine/arginine-rich splicing factor 9
SRSF2: Serine/arginine-rich splicing factor 2
SRSF3: Serine/arginine-rich splicing factor 3
RBFOX2: RNA binding protein fox-1 homolog 2
ROC: Receiver operating characteristics
AUC: Area under curve
EVPL: Envoplakin
