# Supplementary figures and images for: LINC00649 underexpression is an adverse prognostic marker in acute myeloid leukemia
Source: BMC Cancer. 2020 Sep 3;20:841. doi: 10.1186/s12885-020-07331-0 (PMC7469387; doi:10.1186/s12885-020-07331-0)

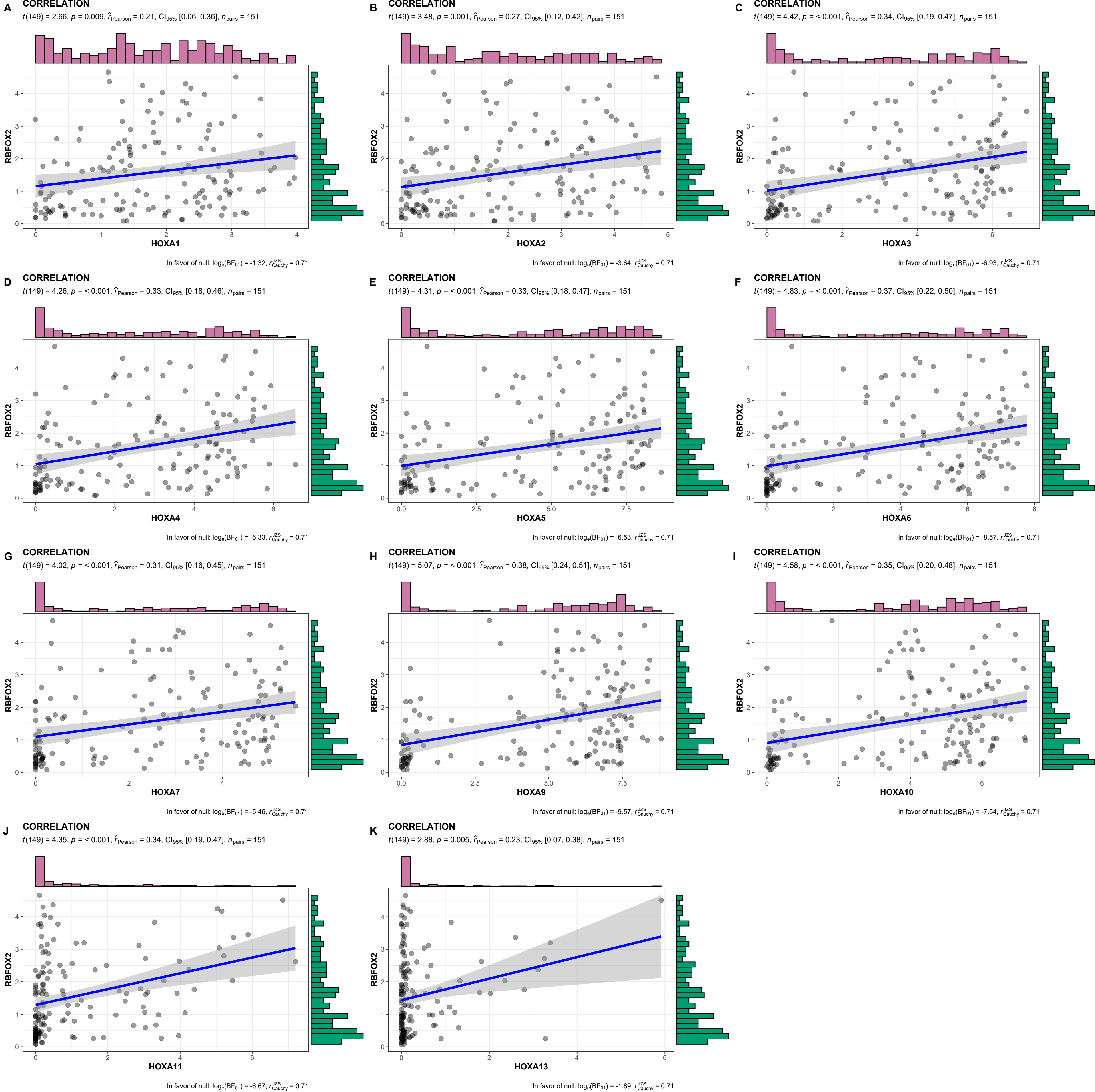

Supplement: Supplementary file 5 — Additional file 5: Figure S1. The results of correlation analysis of RBFOX2 and HOXA genes by R software (version 3.6.0). [file 12885_2020_7331_MOESM5_ESM.pdf]

A

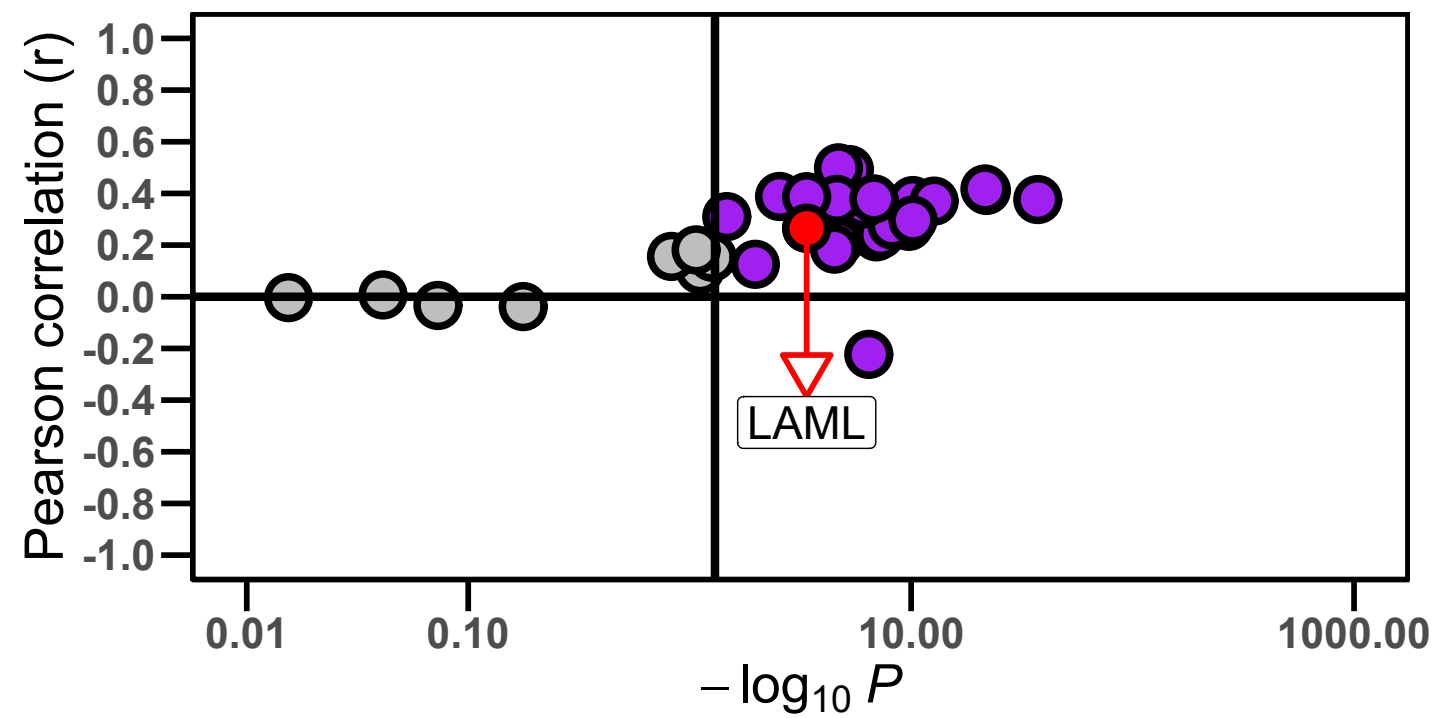

B

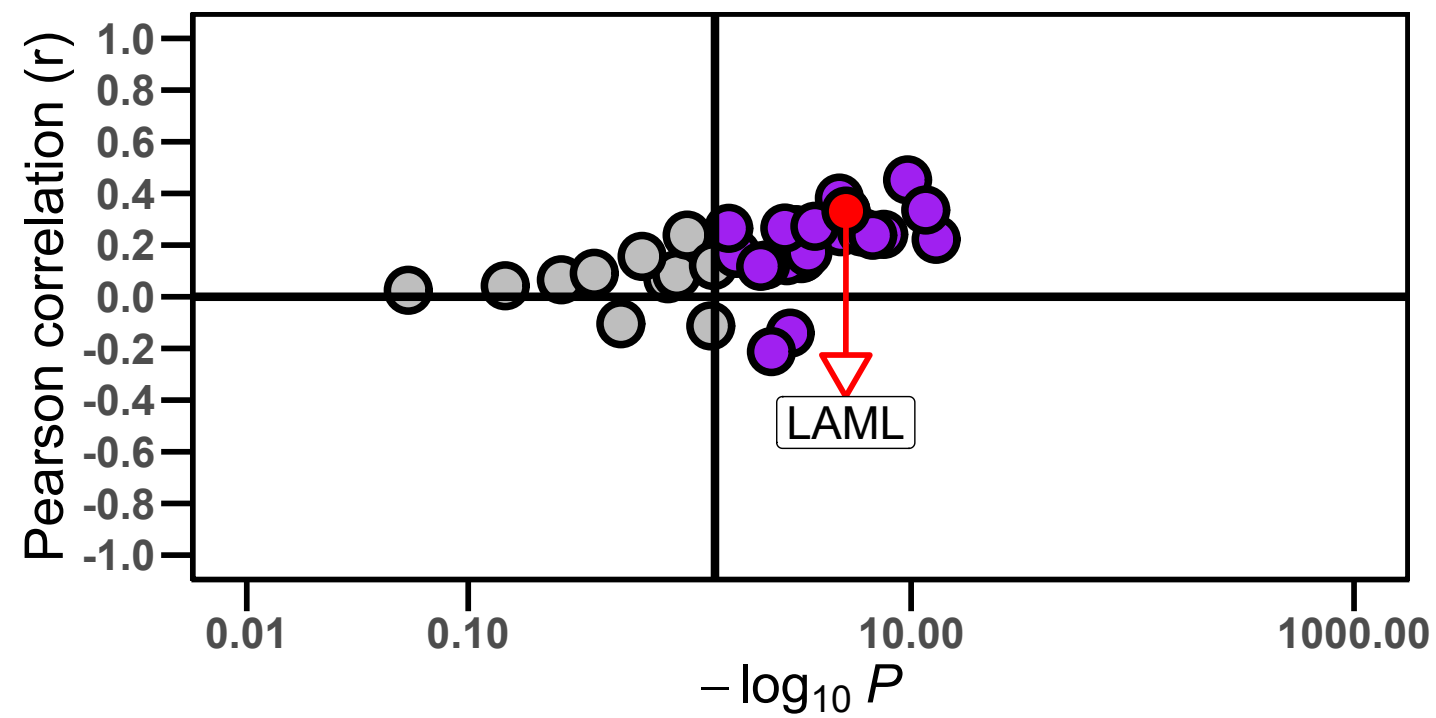

C

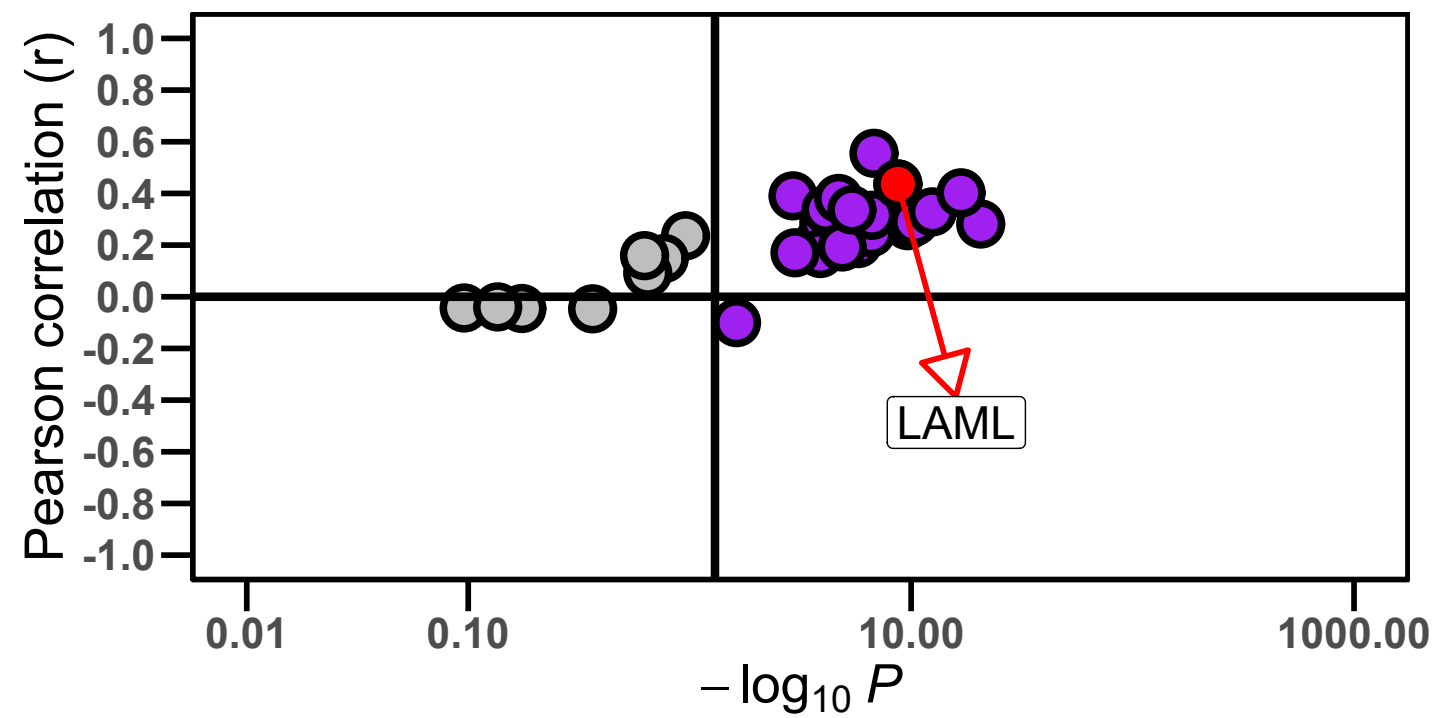

D

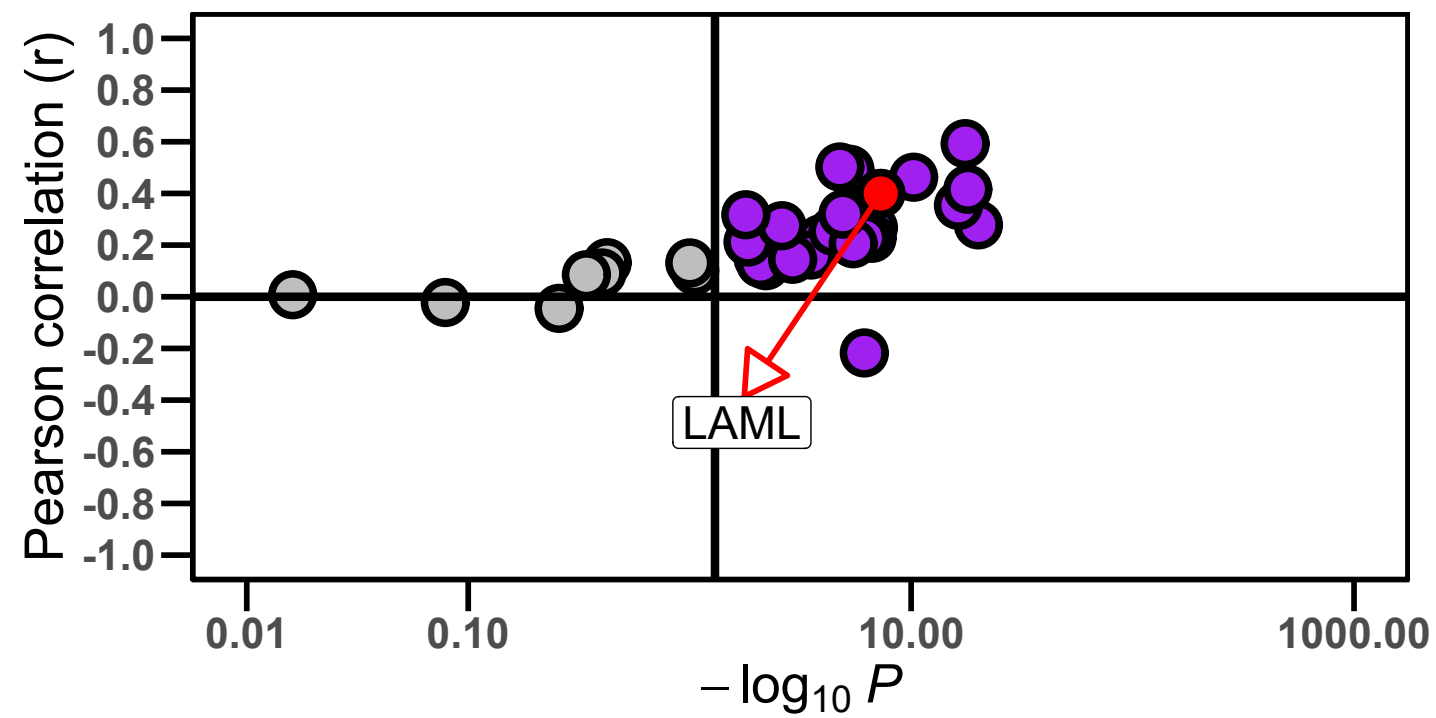

E

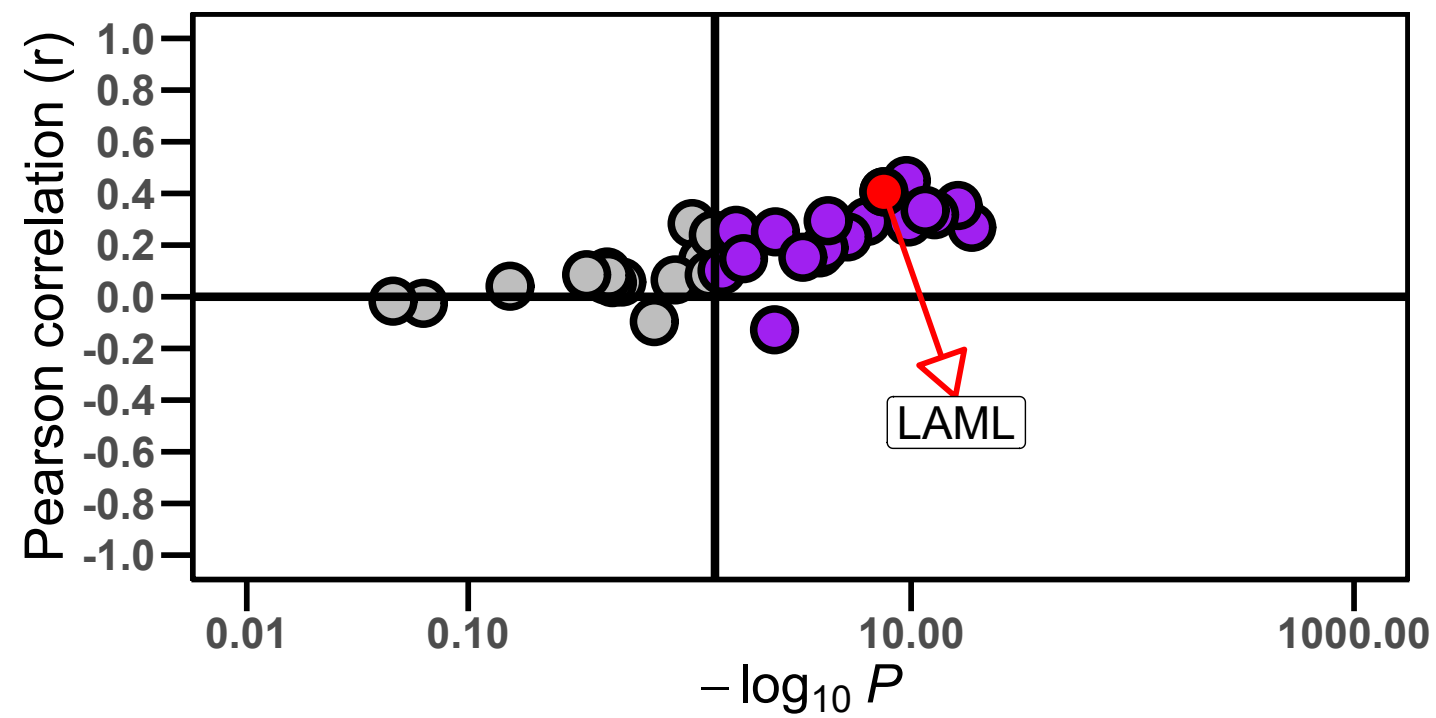

F

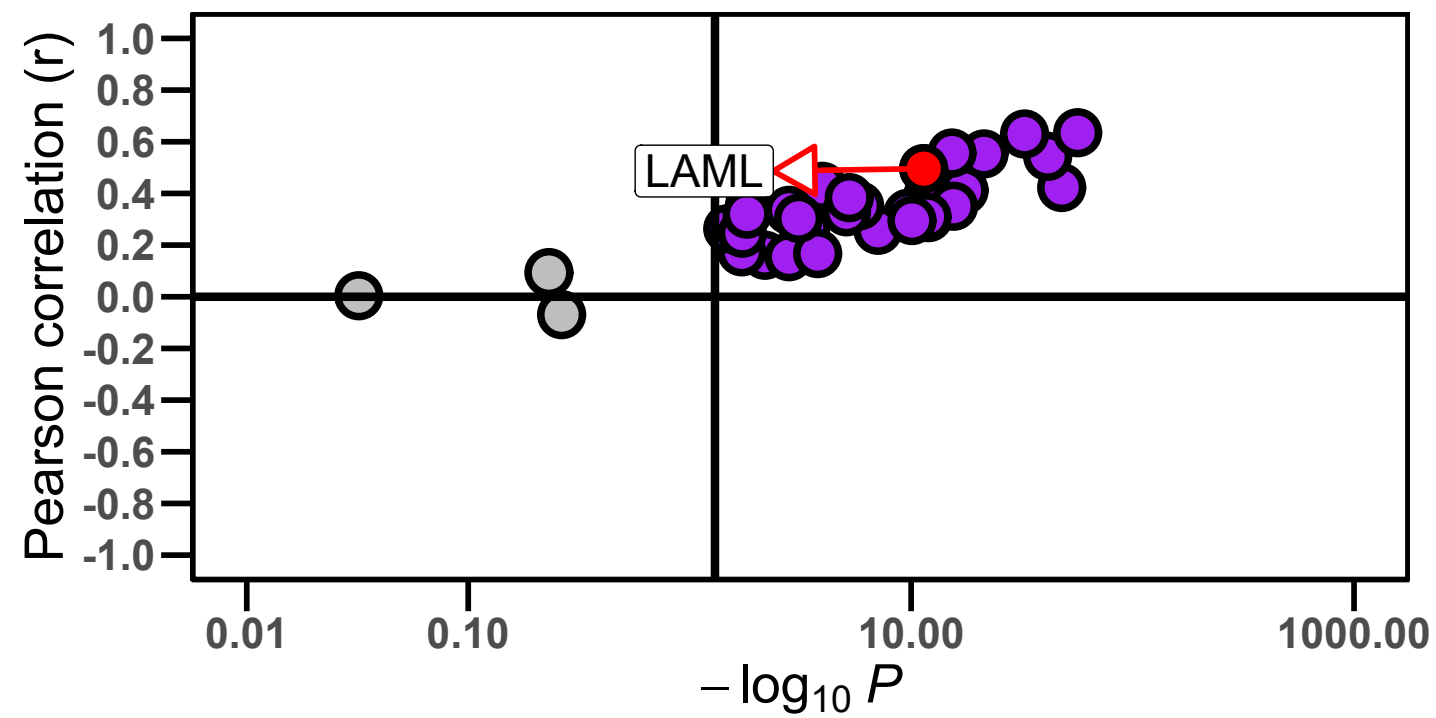

G

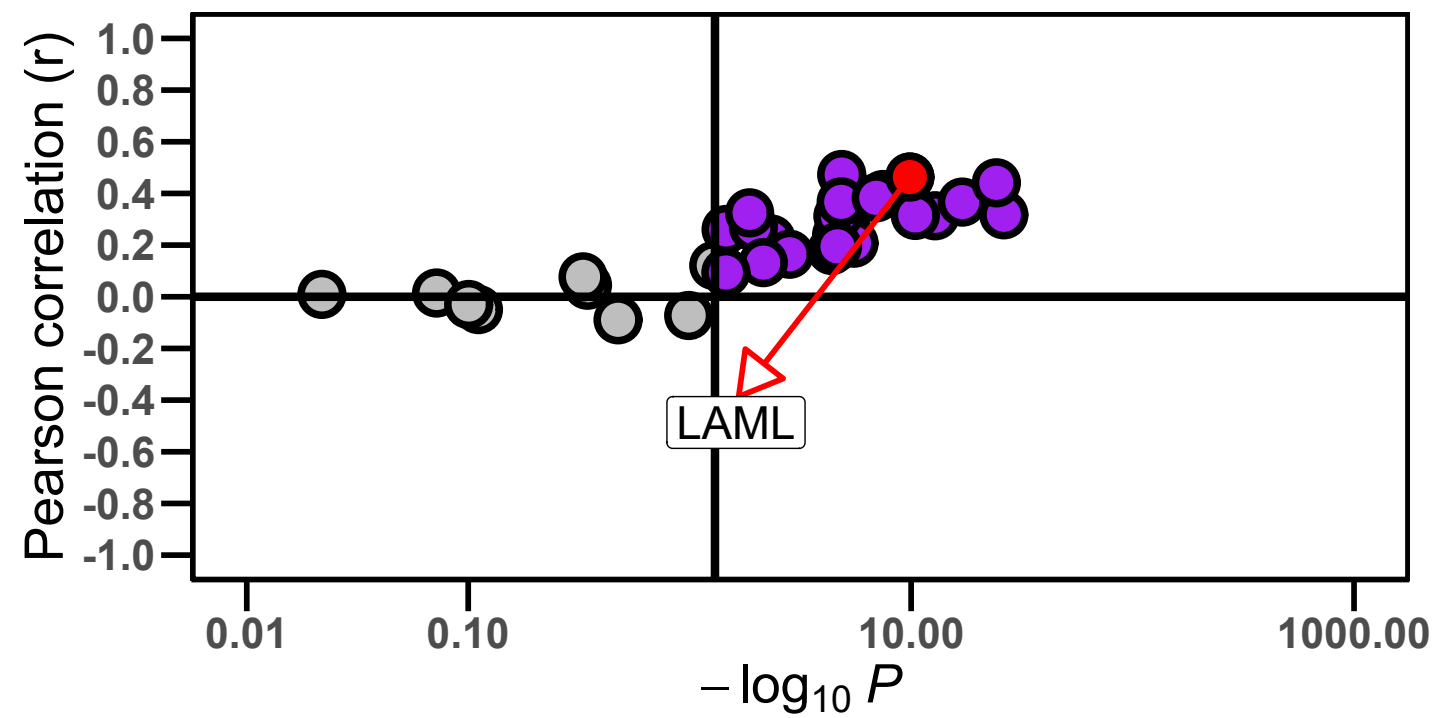

H

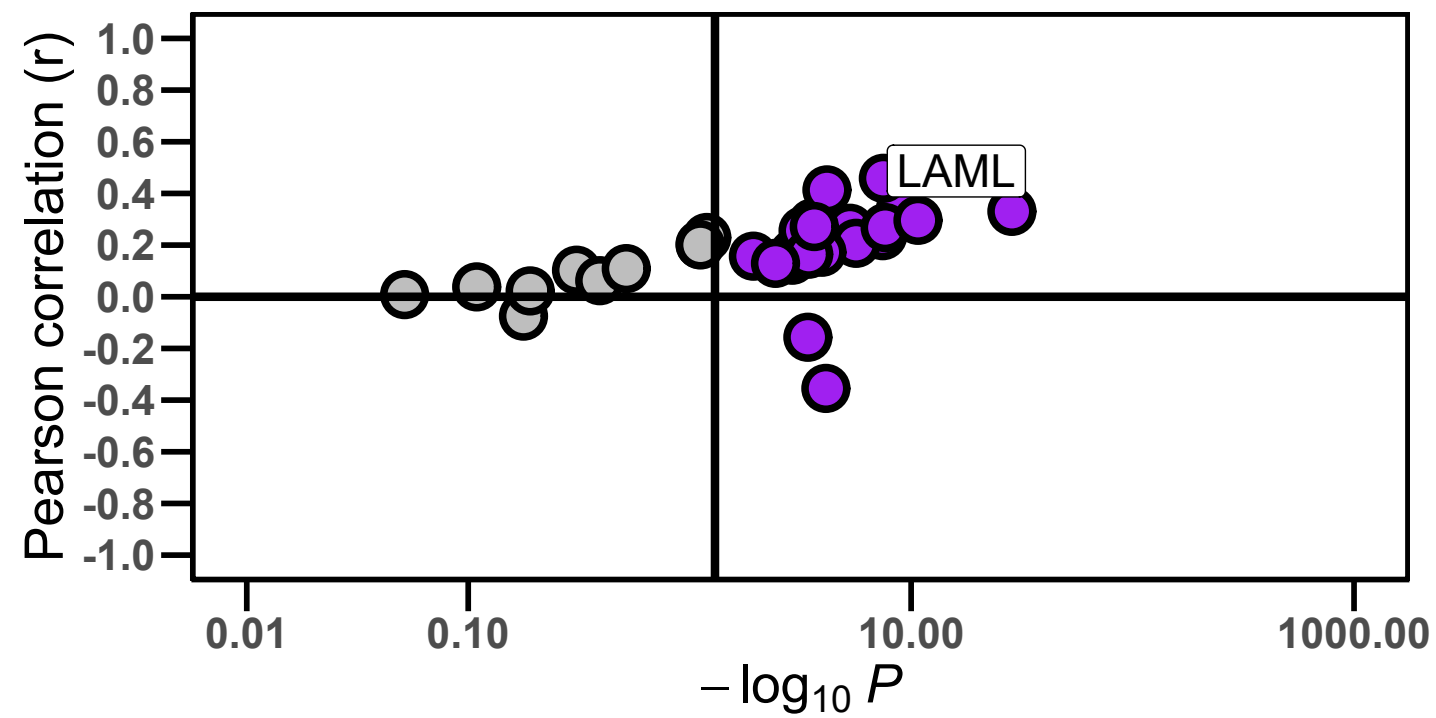

I

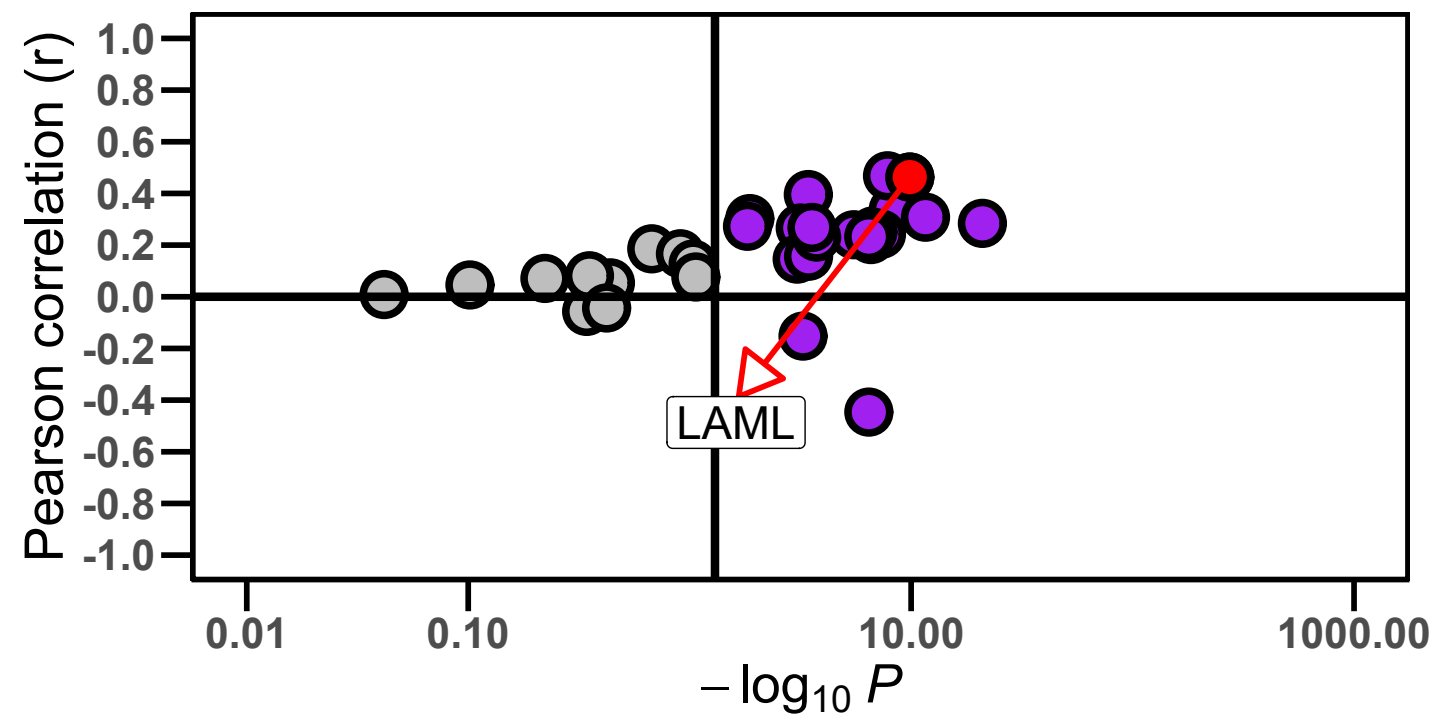

J

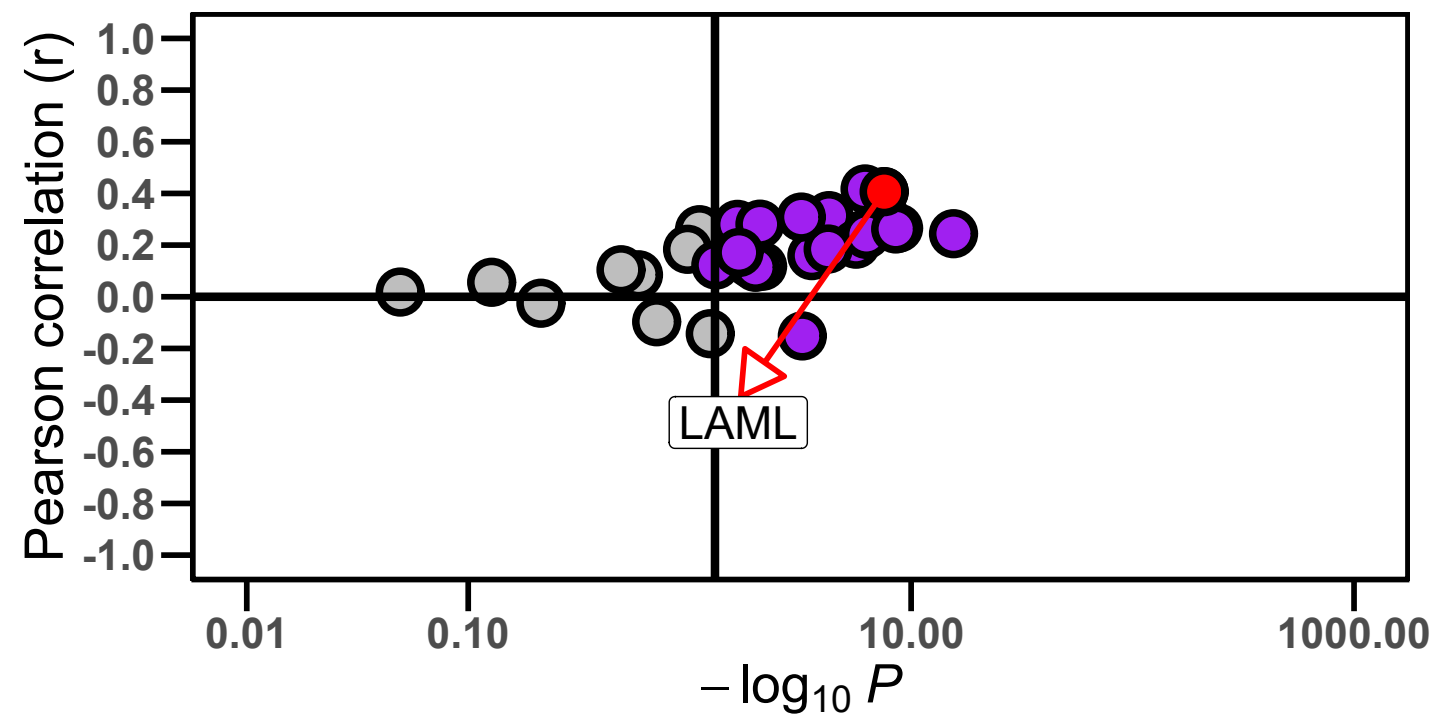

K

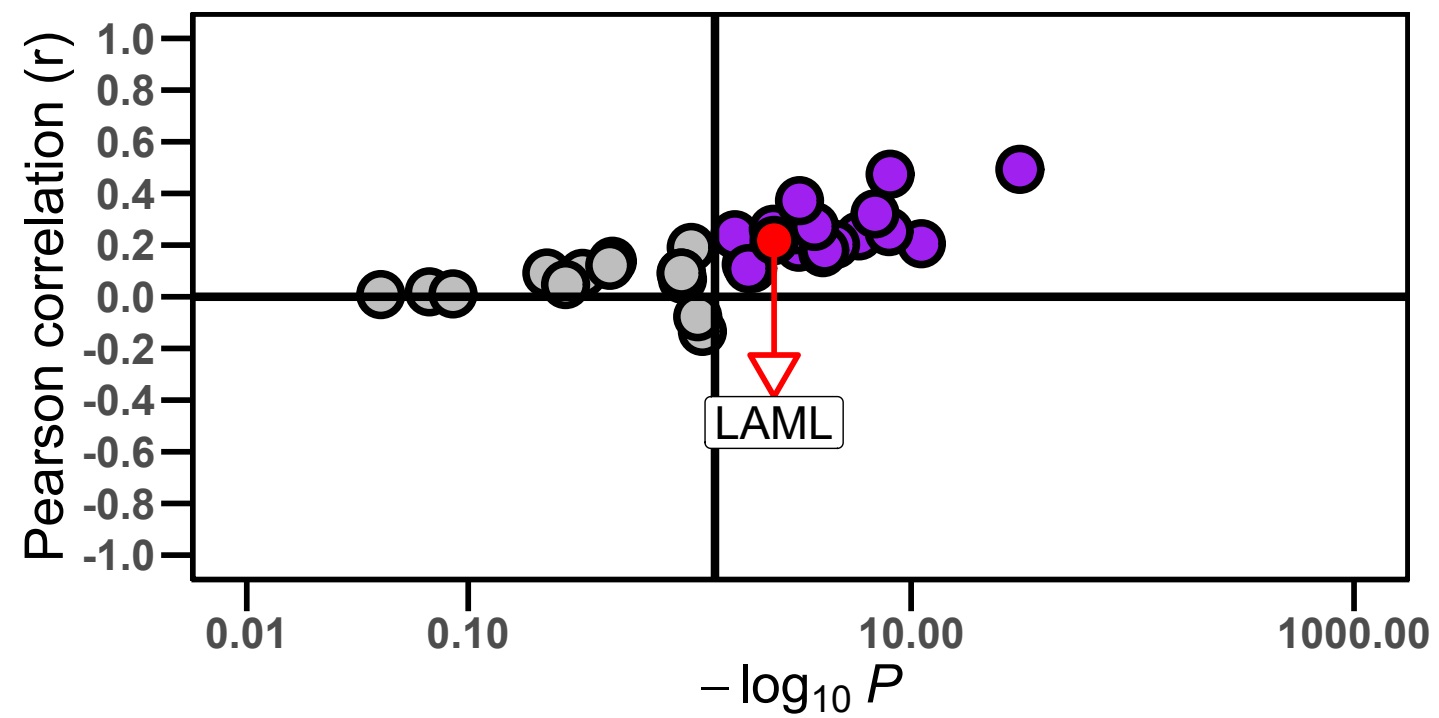

Supplement: Supplementary file 6 — Additional file 6: Figure S2. Pan-cancer correlation analysis of RBFOX2 and HOXA genes by R software (version 3.6.0). The X axis stands for -log10(p value), while the Y axis represent Pearson coefficients. The purple dots in the right upper quadrant represent cancer types, in which the correlation is significant and positive. The result in AML was red dots and annotated by text. [file 12885_2020_7331_MOESM6_ESM.pdf]

A

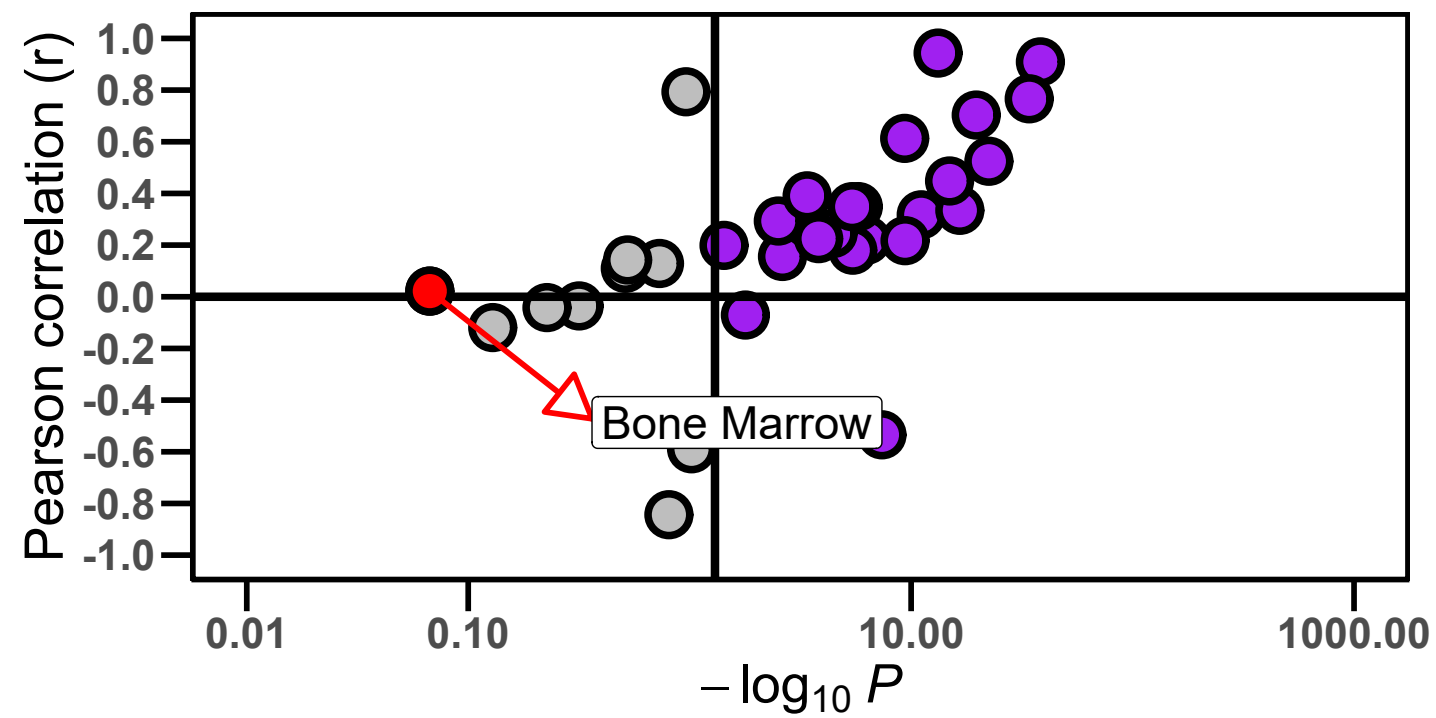

B

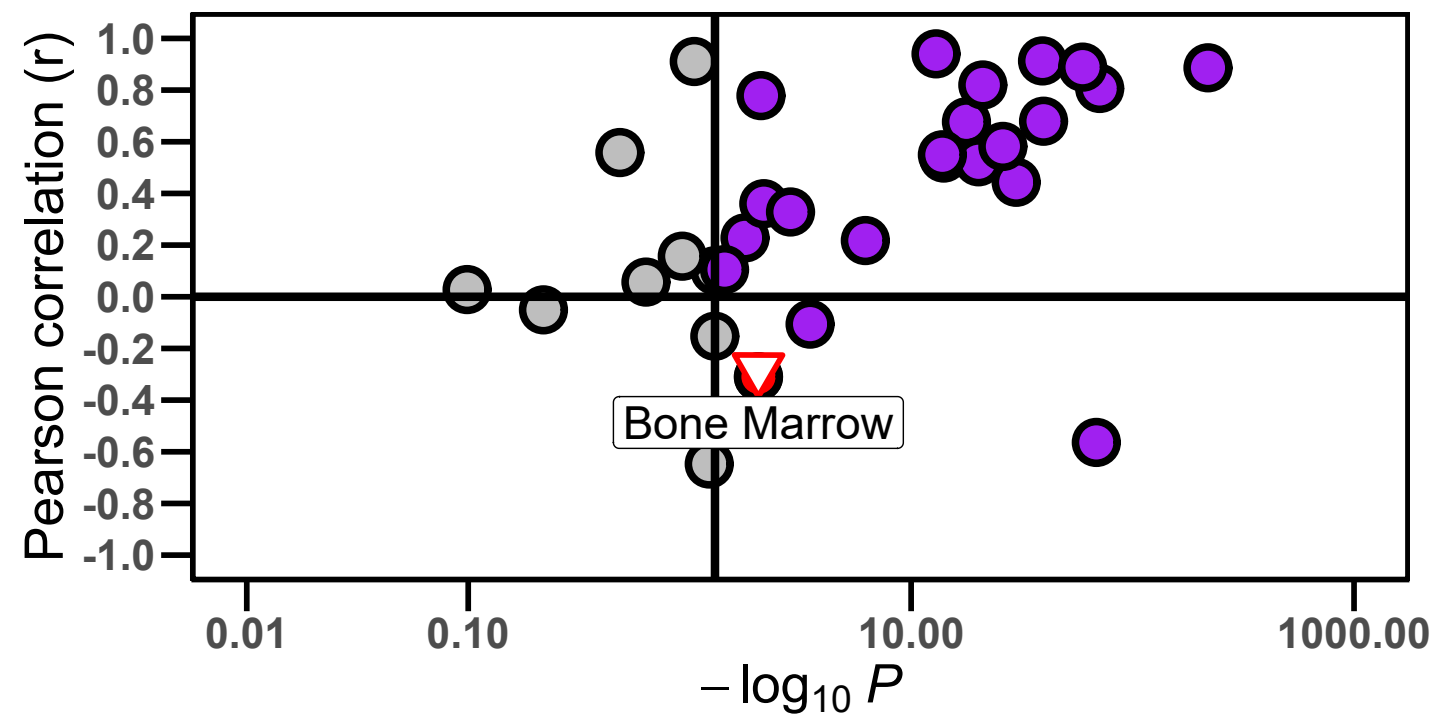

C

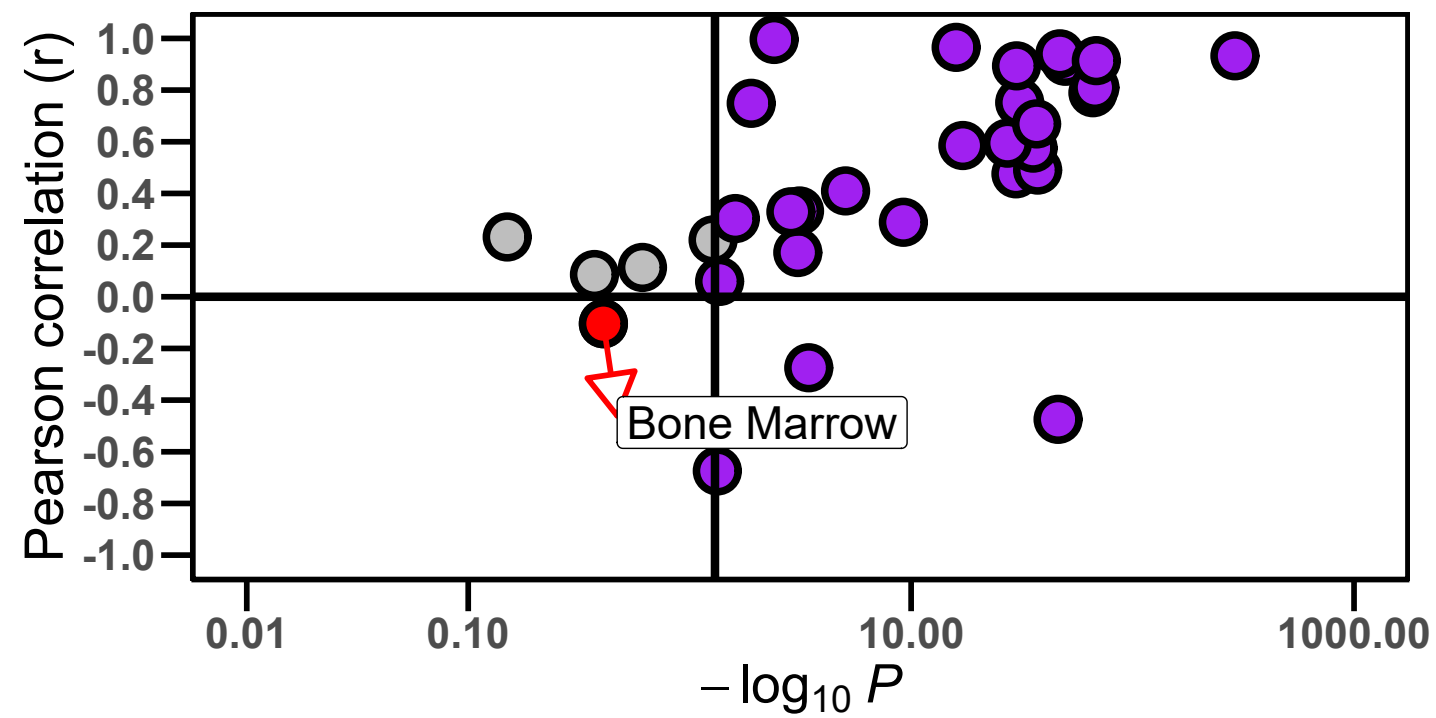

D

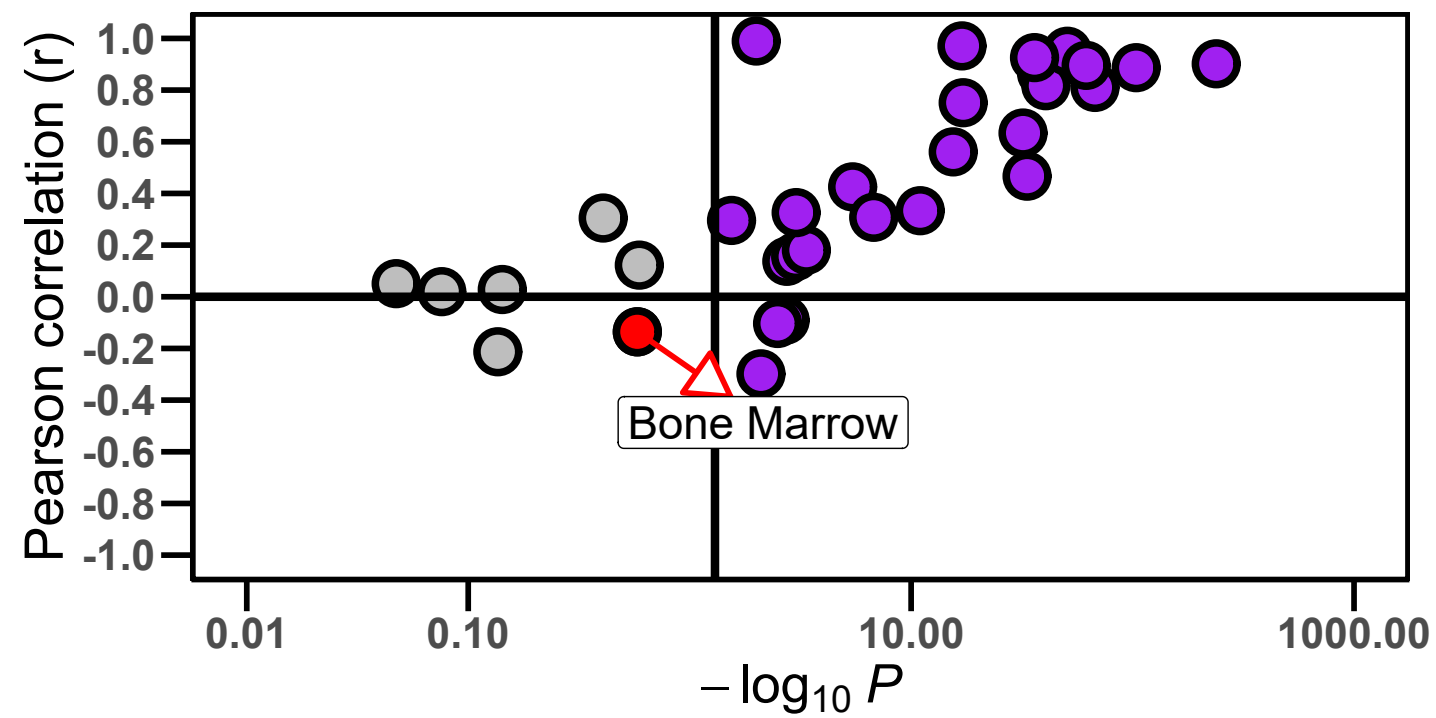

E

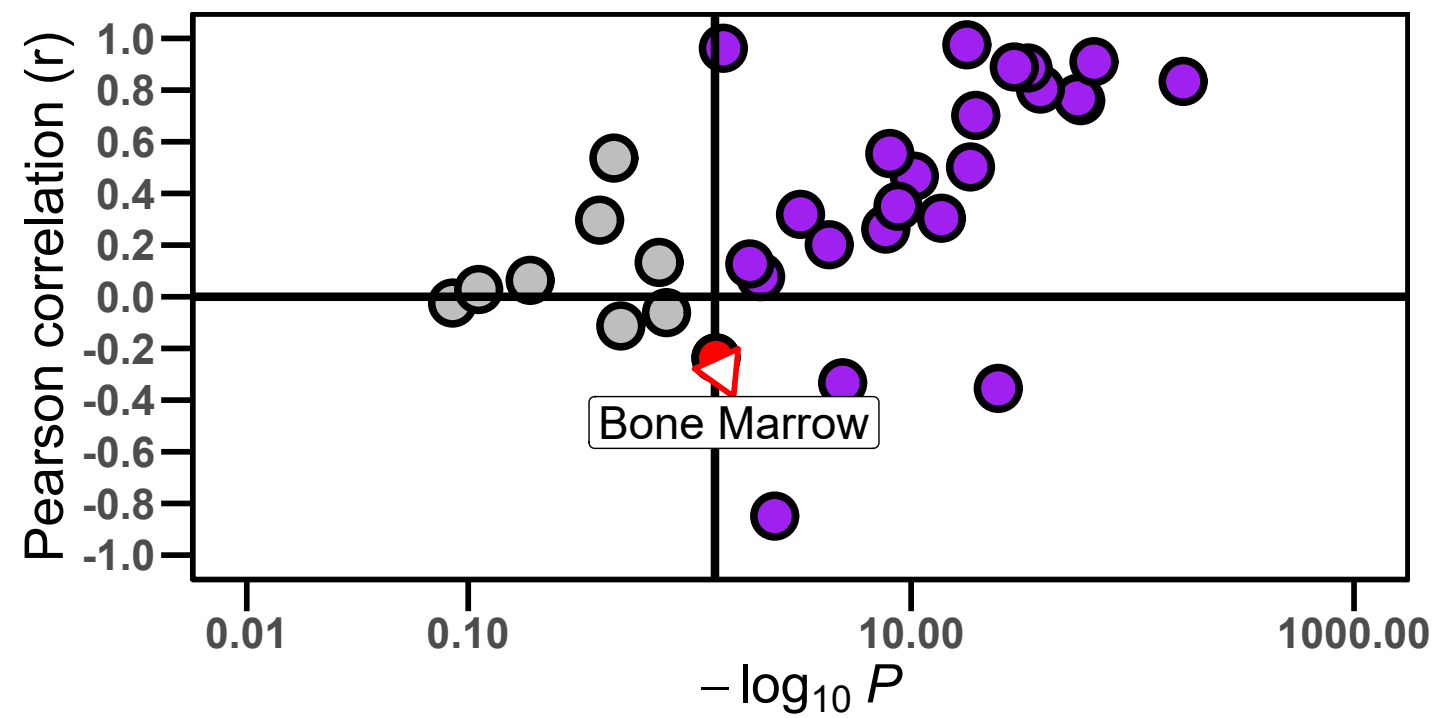

F

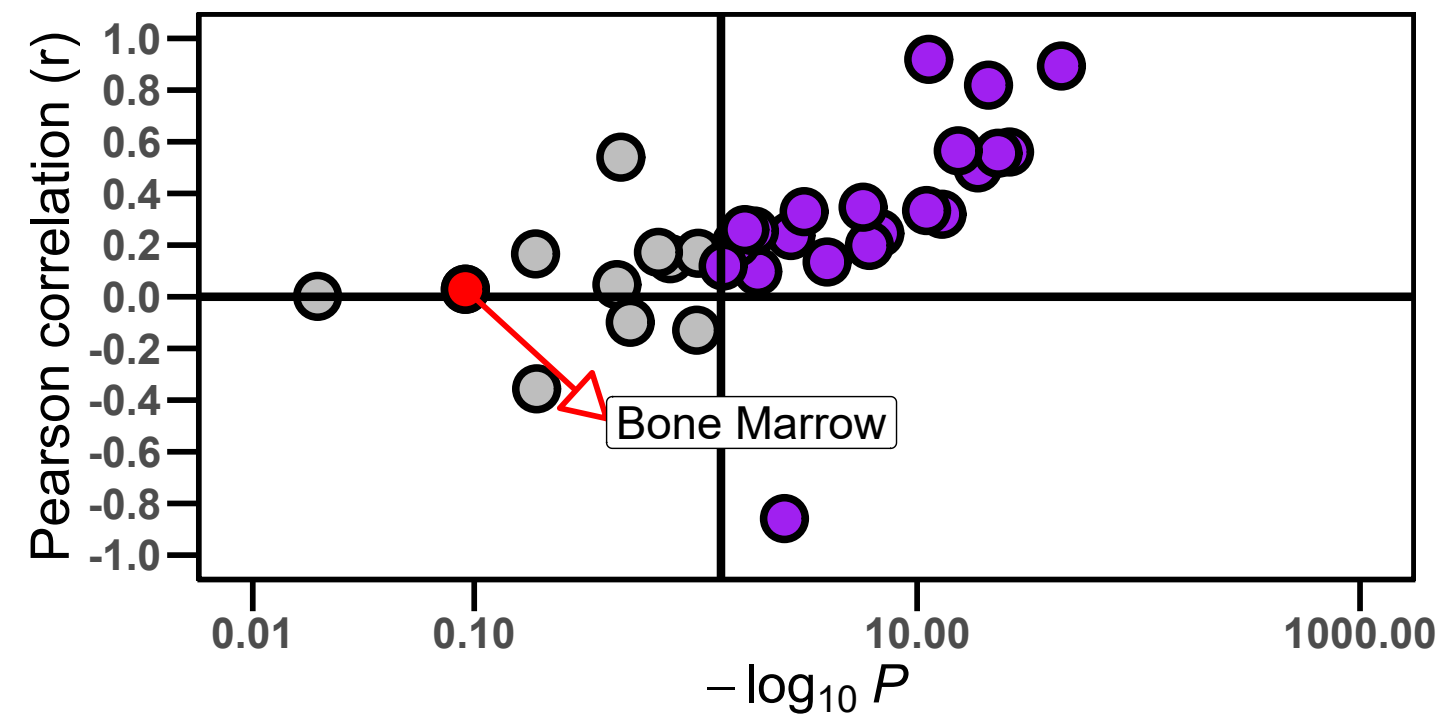

G

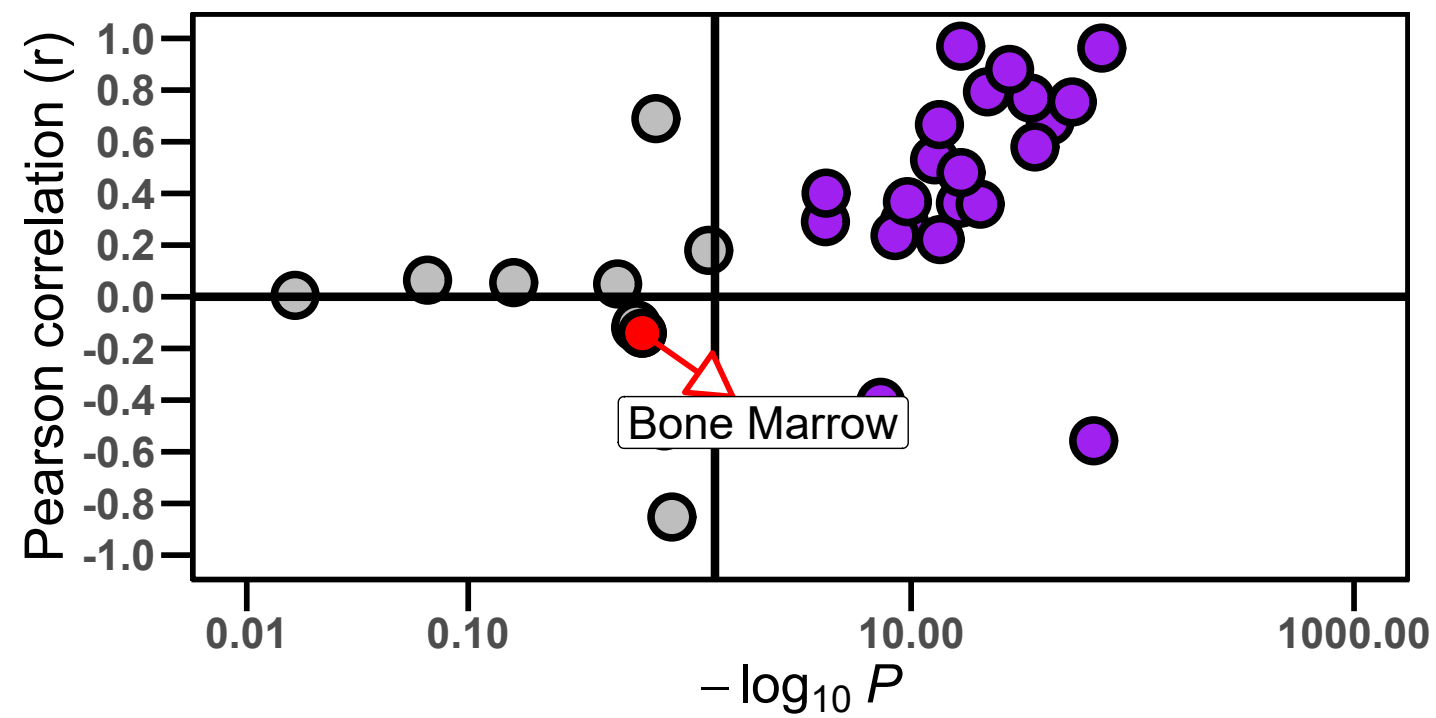

H

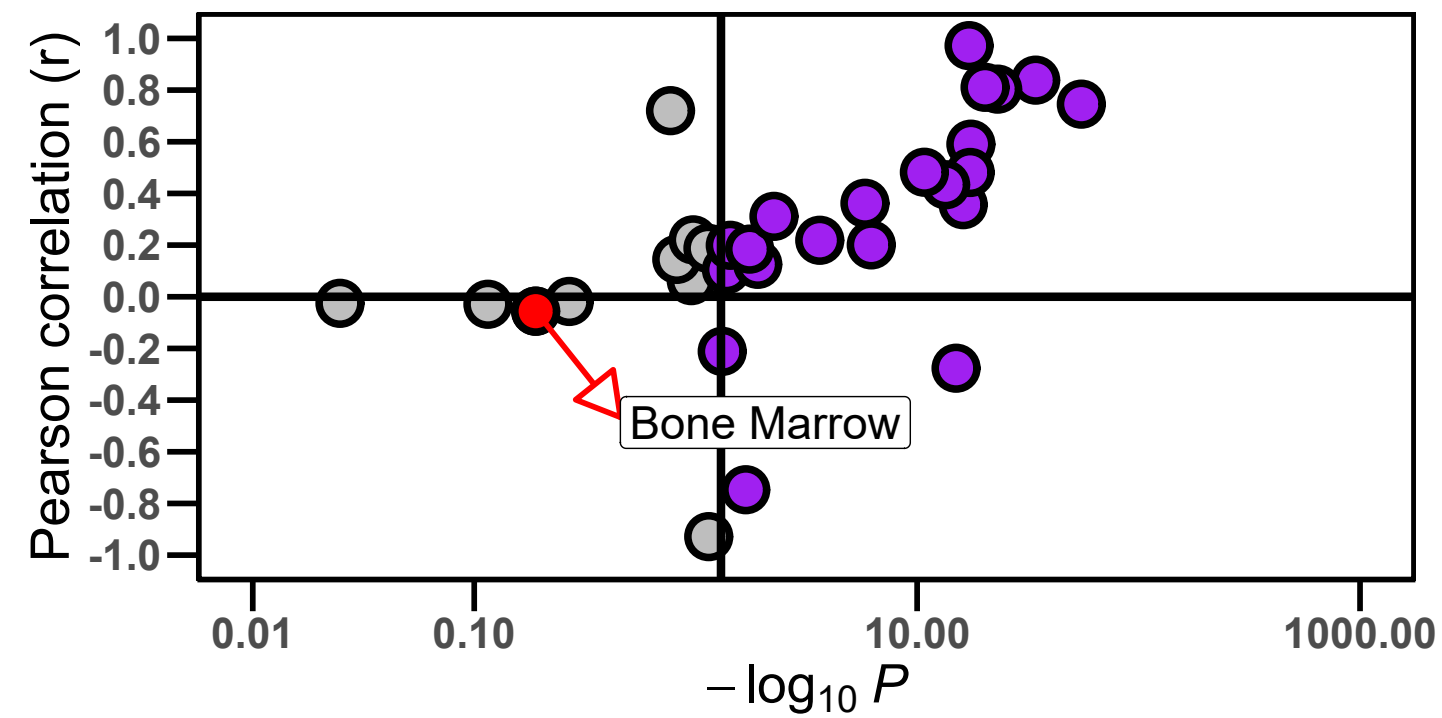

I

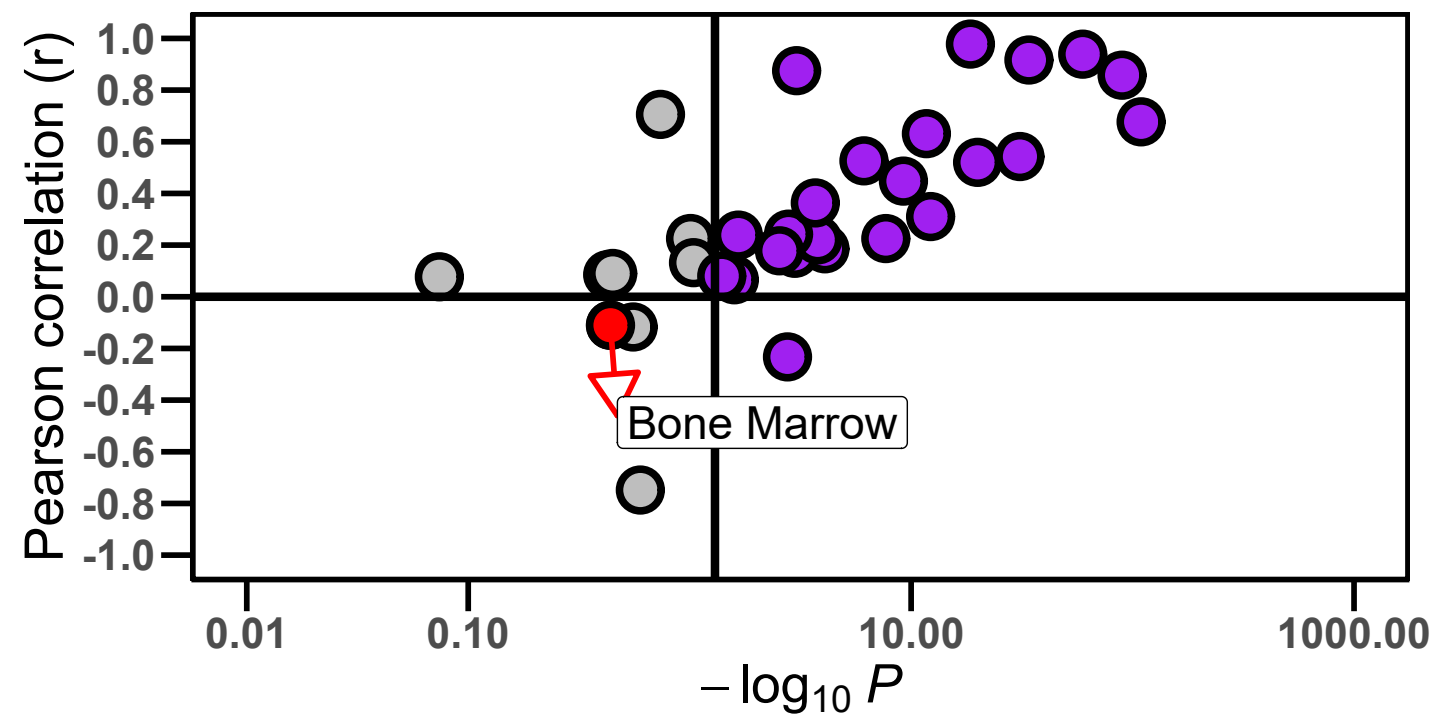

J

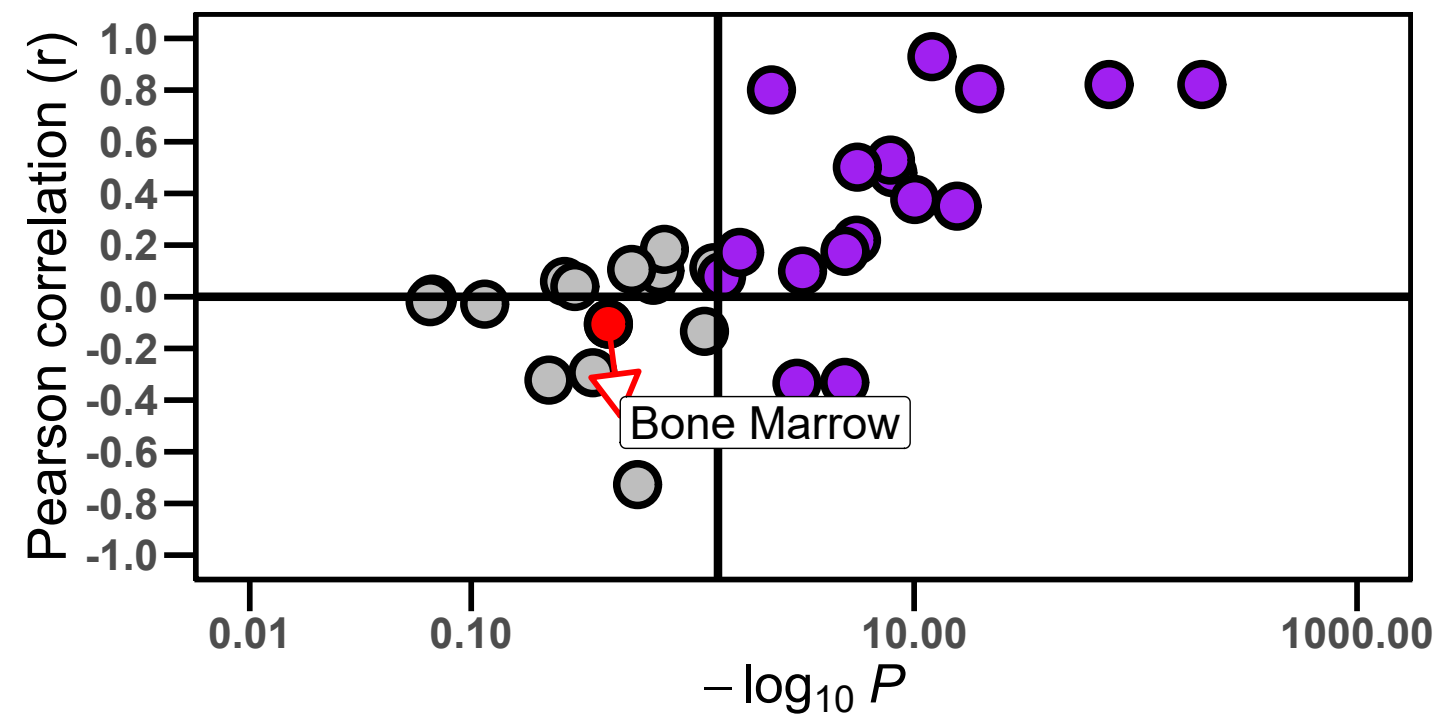

K

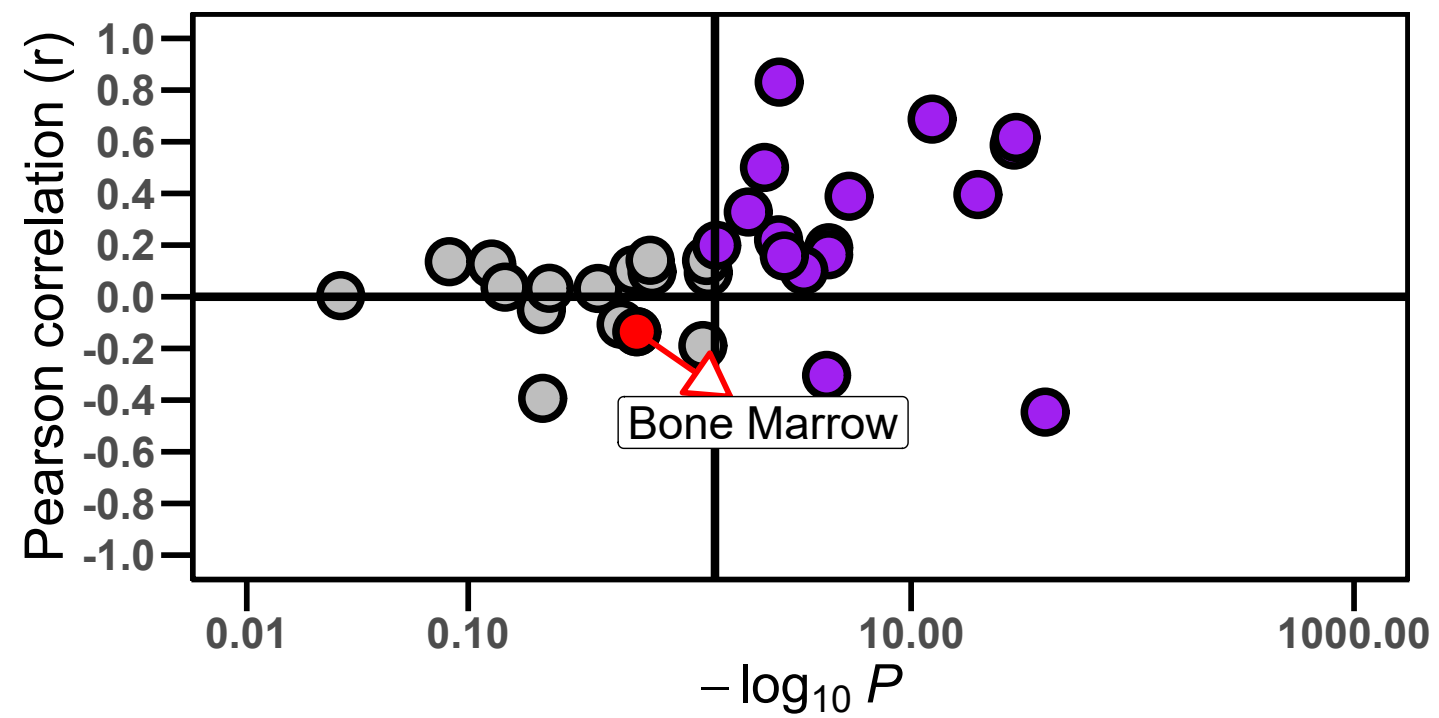

Supplement: Supplementary file 7 — Additional file 7: Figure S3. pan-tissue correlation analysis of RBFOX2 and HOXA genes by R software (version 3.6.0). The X axis stands for -log10(p value), while the Y axis represent Pearson coefficients. The purple dots in the right upper quadrant represent normal tissue types, in which the correlation is significant and positive. The result in normal bone marrow was red dots and annotated by text. [file 12885_2020_7331_MOESM7_ESM.pdf]

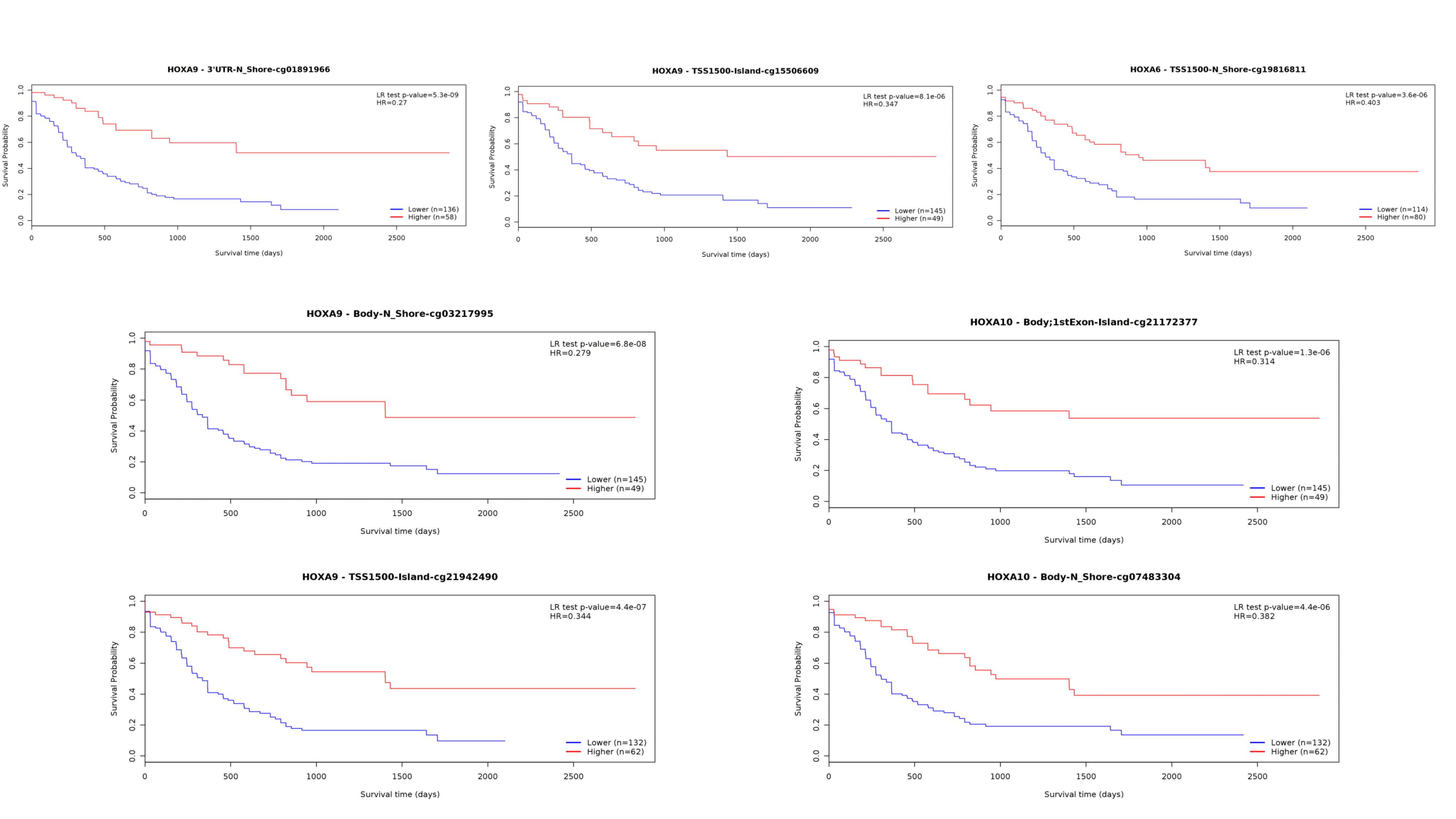

Supplement: Supplementary file 8 — Additional file 8: Figure S4. The results of Kaplan-Meier analysis obtained by MethSurv online database. [file 12885_2020_7331_MOESM8_ESM.jpg]

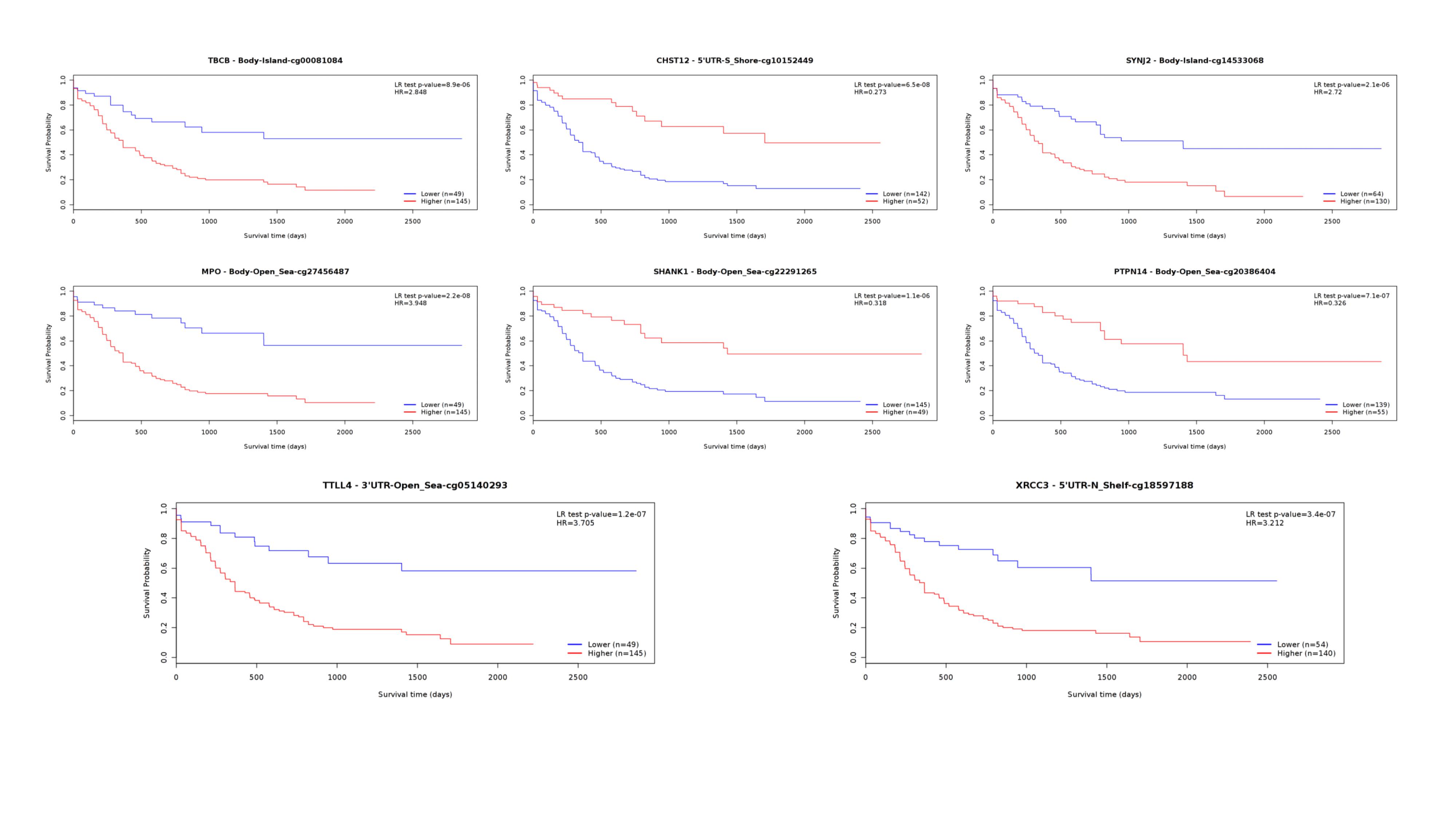

Supplement: Supplementary file 9 — Additional file 9: Figure S5. The results of Kaplan-Meier analysis obtained by MethSurv online database. [file 12885_2020_7331_MOESM9_ESM.jpg]
